# Glucagon-like peptide-1: a critical link between gut microbiota dysbiosis and degenerative musculoskeletal diseases

**DOI:** 10.1080/19490976.2026.2661854

**Published:** 2026-04-22

**Authors:** Wenxing Yang, Cong Hao, Ning Wang, Jie Xie, Bin Zhou, Zidan Yang, Aojie Zheng, Jie Wei, Changjun Li, Cen Xie, Hui Li, Guanghua Lei, Chao Zeng

**Affiliations:** a Department of Orthopaedics, Xiangya Hospital, Central South University, Changsha, People's Republic of China; b Key Laboratory of Aging-related Bone and Joint Diseases Prevention and Treatment, Ministry of Education, Xiangya Hospital, Central South University, Changsha, People's Republic of China; c Hunan Key Laboratory of Joint Degeneration and Injury, Changsha, People's Republic of China; d Department of Epidemiology and Health Statistics, Xiangya School of Public Health, Central South University, Changsha, People's Republic of China; e FuRong Laboratory, Changsha, Hunan, People's Republic of China; f Department of Endocrinology, Endocrinology Research Center, Xiangya Hospital, Central South University, Changsha, People's Republic of China; g State Key Laboratory of Drug Research, Shanghai Institute of Materia Medica, Chinese Academy of Sciences, Shanghai, People's Republic of China; h School of Chinese Materia Medica, Nanjing University of Chinese Medicine, Nanjing, People's Republic of China; i National Clinical Research Center for Geriatric Disorders, Xiangya Hospital, Central South University, Changsha, People's Republic of China

**Keywords:** Degenerative musculoskeletal diseases, gut microbiota, GLP-1

## Abstract

Degenerative musculoskeletal diseases are characterized by the progressive loss of structural integrity and functional capacity in muscles, bones, joints, and other related tissues. These conditions primarily include osteoarthritis, osteoporosis, sarcopenia, and intervertebral disc degeneration. These diseases progress gradually, leading to chronic pain, impaired mobility, and a significantly elevated risk of disability. Given their high prevalence and substantial health burden, degenerative musculoskeletal diseases pose a major challenge in the context of population aging. Although various types of degenerative musculoskeletal diseases affect different primary tissues, they share common pathological features including chronic inflammation, oxidative stress, and dysregulated cell death. These shared mechanisms imply the existence of a common molecular regulatory network. Glucagon-like peptide-1 (GLP-1), a peptide secreted under the regulation of the gut microbiota (GM), has garnered increasing attention for its protective roles in bone, cartilage, muscle, and intervertebral disc tissues. GLP-1 reduces inflammation, mitigates oxidative stress, inhibits apoptosis, and supports microbial homeostasis. This review outlines the mechanisms through which the GM regulates GLP-1 secretion. It further summarizes the core functions and disease-specific pathways by which GLP-1 confers protection against degenerative musculoskeletal diseases. Finally, it explores potential therapeutic strategies targeting the “GM–GLP-1” axis, highlighting microbiota modulation and GLP-1 pathway enhancement as promising treatment approaches for degenerative musculoskeletal diseases.

## 1. Introduction

The rapid aging of the global population has placed degenerative musculoskeletal diseases at the forefront of public health concerns. These conditions—primarily including osteoarthritis (OA), osteoporosis (OP), sarcopenia (SP), and intervertebral disc degeneration (IVDD)—affect hundreds of millions of people worldwide and represent a major cause of pain, disability, and reduced life expectancy among older adults. OA and IVDD each affect over 500 million individuals globally,^1-4^ OP affects approximately 200 million,^5^^,^^6^ and SP impacts an estimated 50 million older adults.^7-9^ The clinical consequences—ranging from joint dysfunction and fragility fractures to physical frailty, impaired mobility, and heightened fall risk^1^^,^^3^^,^^7^^,^^10^—impose a growing burden on healthcare systems and underscore the urgent need for more effective preventive and therapeutic strategies.

Glucagon-like peptide-1 (GLP-1), a peptide hormone secreted by intestinal L cells, exerts systemic effects through the GLP-1 receptor (GLP-1R),^11-13^ a G protein-coupled receptor (GPR) expressed ubiquitously across peripheral tissues.^11^ Although primarily characterized for its regulation of glucose and lipid metabolism,^14^ GLP-1 is increasingly recognized for its protective efficacy in degenerative musculoskeletal diseases.^15-18^ Mechanistically, GLP-1 signaling mitigates tissue degeneration by modulating nuclear factor kappa B (NF-κB),^19^ reactive oxygen species (ROS),^20^ and B-cell lymphoma 2 (Bcl-2) pathways,^21^ thereby driving critical anti-inflammatory, antioxidant, and anti-apoptotic processes.

While the secretion of GLP-1 is canonically regulated by a multitude of factors, including nutrient ingestion (e.g., glucose, lipids),^22^^,^^23^ neural reflexes (e.g., vagus nerve),^24^^,^^25^ and hormonal signals,^26^^,^^27^ the gut microbiota (GM) assumes a preeminent regulatory role in the context of musculoskeletal pathology.^28^^,^^29^ This specific relevance arises from the physiology of distal L cells in the ileum and colon, where bacterial density peaks.^28^^,^^30^ Unlike their proximal counterparts stimulated by direct nutrient contact, these distal cells rely heavily on microbial metabolites—such as short-chain fatty acids (SCFAs) and secondary bile acids (BAs)—because most dietary nutrients are absorbed upstream.^25^^,^^30-32^ Consequently, the gut dysbiosis associated with aging and degenerative musculoskeletal diseases impairs this metabolite-driven stimulation, resulting in GLP-1 deficiency that is not primarily regulated by diet.^33^^,^^34^ Experimentally, depleting gut bacteria (via antibiotics or in germ‑free mice) abolishes postprandial GLP‑1 in circulation and ileum, while fecal microbiota transplantation (FMT) restores secretion—directly demonstrating the microbiota’s necessary role.^35^ Thus, although neural/hormonal signals contribute, the microbiota is fundamental for meal‑induced GLP‑1 release. Crucially, unlike inaccessible neural circuits or diffuse hormonal networks, the GM is a readily actionable target, modifiable through prebiotics, probiotics, or FMT. Therefore, targeting the “GM–GLP-1” axis represents a strategy to restore physiological GLP-1 signaling.

**Figure 1.**
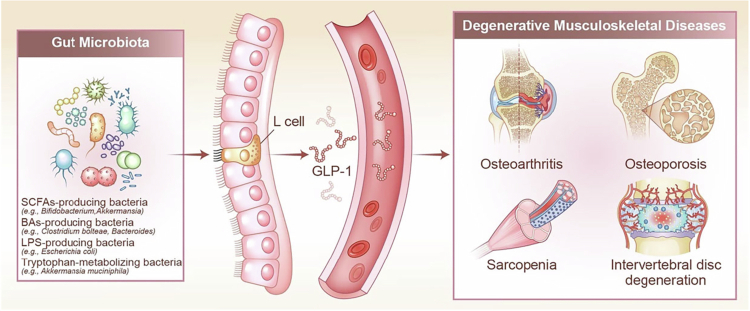
Schematic summary illustrating how gut microbiota influences degenerative musculoskeletal diseases through GLP-1 signaling. The gut microbiota interacts with intestinal L cells to stimulate the secretion of GLP-1, which in turn affects the progression of degenerative conditions such as osteoarthritis, osteoporosis, sarcopenia, and intervertebral disc degeneration. The key microbiota groups promoting GLP-1 secretion include: 1. SCFAs-producing bacteria, which generate SCFAs to activate GPR41/43 on L cells. 2. BAs-producing bacteria, which metabolize primary bile acids into secondary forms that act as TGR5 agonizts or FXR antagonists. 3. LPS-producing bacteria, whose LPS can modulate GLP-1 release through TLR4 signaling. 4. Tryptophan-metabolizing bacteria, which convert dietary tryptophan into indole derivatives and other metabolites that activate host receptors such as GPR142. GLP-1: glucagon-like peptide-1; SCFAs: short-chain fatty acids; GPR: G protein-coupled receptor; BAs: bile acids; TGR: takeda G protein-coupled receptor; FXR: farnesoid X receptor; LPS: lipopolysaccharides; TLR: Toll-like receptor.

This review focuses on the mechanisms through which GM influences GLP-1 secretion and the role of GLP-1 in the progression of degenerative musculoskeletal diseases. We first examine the microbial pathways regulating GLP-1 biosynthesis, followed by a summary of GLP-1's downstream effects and impact on pathological changes across different tissues in degenerative musculoskeletal diseases. Finally, we discuss the translational potential of targeting the “GM–GLP-1” axis as a therapeutic strategy for treating degenerative musculoskeletal diseases in aging populations (Figure 1).

## 2. Molecular mechanisms of GM signaling regulating GLP-1

**Figure 2.**
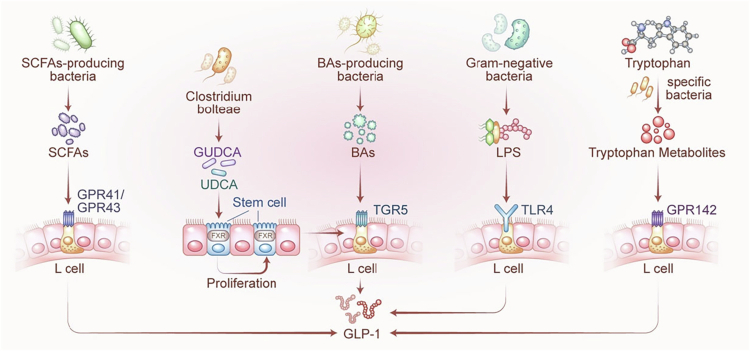
Mechanisms through which gut microbiota regulate GLP-1 secretion via L cells. SCFAs bind to GPR41 and GPR43 receptors on L cells, enhancing GLP-1 secretion. BAs activate the TGR5 receptor on L cells, increasing GLP-1 secretion. *Clostridium bolteae* produces GUDCA and UDCA, which blocks FXR signaling, thereby promoting the proliferation of intestinal stem cells, increasing L cell numbers, and enhancing GLP-1 secretion. LPS released by Gram-negative bacteria activates TLR4 receptors on L cells, further boosting GLP-1 secretion. Tryptophan metabolites interact with GPR142 receptors on L cells, modulating their function and promoting additional GLP-1 release. These pathways collectively play a crucial role in regulating GLP-1 secretion through the intricate interactions between microbiota-derived metabolites and L cells. GLP-1: glucagon-like peptide-1; SCFAs: short-chain fatty acids; GPR: G protein-coupled receptor; BAs: bile acids; TGR: takeda G protein-coupled receptor; GUDCA: glycoursodeoxycholic acid; UDCA: ursodeoxycholic acid; FXR: farnesoid X receptor; LPS: lipopolysaccharides; TLR: Toll-like receptor.

GM generates a wide array of bioactive small molecules through the metabolism of dietary components. These microbial metabolites function as critical mediators linking the intestinal ecosystem to host physiology.^36^ Among various host–microbiota interactions, the regulation of enteroendocrine L cells and their secretion of GLP-1 represents a key mechanism by which GM influences systemic energy balance and tissue homeostasis.^30^ Beyond its well-established role in glycemic control,^37^^,^^38^ GLP-1 has gained attention for its involvement in bone remodeling,^39^ muscle protein synthesis,^40^ and anti-inflammatory responses.^41^^,^^42^ Notably, impaired GLP-1 signaling is increasingly implicated in the pathogenesis of degenerative musculoskeletal diseases. Elucidating how microbial metabolites regulate GLP-1 production is therefore essential for the development of novel therapeutic strategies targeting these conditions. In the following sections, we detail the specific roles and underlying mechanisms of SCFAs, BAs, lipopolysaccharides (LPS), and tryptophan-derived metabolites within the GM–metabolite–L cell–GLP-1 signaling axis (Figure 2).

### 2.1 SCFAs-mediated GLP-1 regulation

SCFAs (C1–C6 fatty acids) are key microbial metabolites derived from the fermentation of indigestible carbohydrates such as dietary fiber, oligosaccharides, *β*-glucan, and chitin. Acetate (~60%), propionate (~25%), and butyrate (~15%) are the predominant SCFAs, together accounting for over 95% of total SCFAs in the healthy human colon, where their concentrations exceed 100 millimolar (mM).^31^^,^^43^ In addition to serving as energy substrates for colonocytes, SCFAs act as potent signaling molecules that modulate GLP-1 secretion from intestinal L cells through both direct and indirect mechanisms.^31^^,^^44-47^ Their steep concentration gradient from the intestinal lumen to the circulation enables localized paracrine signaling in the gut microenvironment.^48-50^

A variety of GM taxa play a crucial role in SCFAs production, thereby influencing GLP-1 release. For instance, *fructooligosaccharides* (a common prebiotic) promote the growth of *Bifidobacterium* and *Lactobacillus*,^51^ both key genera involved in SCFAs synthesis. Similarly, polysaccharides from Achyranthes bidentata (a traditional Chinese herb) enhance the abundance of *Prevotellaceae*_UCG_001, *Bacteroides*, *Prevotella*, and *Akkermansia*,^52^ microbes whose population shifts indirectly regulate SCFAs production. Polysaccharides extracted from fingered citron (a citrus fruit) upregulate *Prevotella*, *Lachnospiraceae*, and *Lactobacillaceae*,^53^ with *Lachnospiraceae* being a major family responsible for SCFAs generation in the gut. In addition, resistant starch (a non-digestible carbohydrate abundant in whole grains and legumes) enriches *Bifidobacterium*, *Ruminococcus*, and *Lachnospira*, while enhancing propionate and butyrate levels—important for maintaining gut barrier function and providing energy to intestinal epithelial cells.^54^ Composite probiotics, containing multiple beneficial bacteria, stimulate the proliferation of *Lactobacillus*, *Bifidobacterium*, and *Roseburia* (a key butyrate-producing genus), directly boosting SCFAs production.^55^ Moreover, an almond-based low-carbohydrate diet increases the relative abundance of *Roseburia*, *Ruminococcus*, and *Eubacterium*,^56^ all of which are closely associated with SCFAs metabolism. Isatis root granules (a traditional Chinese medicine) elevate the levels of *Akkermansia* and *Prevotellacea*e_UCG_001, along with increased SCFAs concentrations.^57^ Additionally, *Clostridium* species have been linked to both propionate production and the regulation of GLP-1 secretion,^57^ further reinforcing the “GM–SCFAs–GLP-1” metabolic regulatory network.

Mechanistically, SCFAs regulate GLP-1 release through several interrelated mechanisms. SCFAs activate GPRs, such as free fatty acid receptor 2 (FFAR2) and free fatty acid receptor 3 (FFAR3) (also known as GPR43 and GPR41), on L cells. Particularly, the Gq-coupled FFAR2 triggers an influx of intracellular calcium ions (Ca²⁺), which promotes GLP-1 exocytosis;^31^^,^^46^^,^^51^^,^^57^ this effect is diminished in FFAR2-deficient models.^46^ Additionally, SCFAs suppress inflammatory cytokines like tumor necrosis factor-alpha (TNF-*α*) and interleukin-1 beta (IL-1β), creating an environment that supports GLP-1 release.^52^^,^^55^^,^^57^ SCFAs metabolism also contributes to ATP production, which leads to membrane depolarization and further Ca²⁺ influx.^58^ Furthermore, mitogen-activated protein kinase (MAPK) signaling (*p*-ERK, *p*-p38) enhances the expression of FFARs and GLP-1R.^59^ Negative regulation via the farnesoid X receptor (FXR) inhibits FFAR2 expression, suppressing SCFAs-induced GLP-1 release.^60^ Notably, enterochromaffin (EC) cells detect GLP-1 via GLP-1R and release 5-hydroxytryptamine (5-HT), contributing to neuroendocrine crosstalk through vagal signaling.^59^

Although SCFAs are generally known to act as ligands for the G protein‑coupled receptors FFAR2 and FFAR3 on enteroendocrine L‑cells, their production and their role in regulating GLP‑1 secretion depend largely on the composition of the GM. Unlike acetate, which is generated by many bacterial groups, propionate and butyrate are produced through more phylogenetically restricted pathways. Propionate arises mainly from *Bacteroidetes* species—such as *Bacteroides* and *Prevotella*—via the succinate pathway, and from *Akkermansia muciniphila* through the propanediol pathway.^61^ Butyrate production, in contrast, is mostly carried out by specific members of the Firmicutes phylum, notably *Faecalibacterium prausnitzii* and *Roseburia intestinalis*, which employ the butyryl‑CoA:acetate CoA‑transferase pathway.^62-64^ This metabolic specialization influences how effectively each SCFAs stimulates GLP‑1 secretion. Both propionate and butyrate activate free fatty acid receptors, but propionate shows particularly high efficacy at FFAR2, the primary receptor linked to GLP‑1 release via Gq‑coupled signaling.^31^ Moreover, their physiological availability to L‑cells differs: butyrate is heavily metabolized by colonocytes as an energy source, potentially reducing its basolateral exposure compared with propionate, which reaches the portal circulation more readily.^65^^,^^66^ Consistent with this, extracts from propionate‑producing *A. muciniphila* induce a strong, dose‑dependent increase in GLP‑1 secretion in human NCI‑H716 cells, exceeding the effects of other SCFAs mixtures.^67^^,^^68^ Thus, variations in the *Firmicutes*‑to‑*Bacteroidetes* ratio or in the abundance of key propionate‑producing bacteria may shape incretin responses independently of total SCFAs levels.

### 2.2 BAs-mediated GLP-1 regulation

BAs are amphipathic steroid molecules synthesized from cholesterol in the liver and classified as primary BAs—such as cholic acid (CA), chenodeoxycholic acid (CDCA), and α/β-muricholic acid (MCA) in mice—and secondary BAs, including lithocholic acid (LCA), deoxycholic acid (DCA), ursodeoxycholic acid (UDCA), ωMCA, and hyodeoxycholic acid (HCA). Primary BAs are conjugated with glycine or taurine and secreted into the intestinal lumen. Approximately 95% are reabsorbed in the ileum via the apical sodium-dependent bile acid transporter (ASBT), re-entering the enterohepatic circulation, while the remaining ~5% reach the colon, where they are biotransformed into secondary BAs by the GM through dehydroxylation, deconjugation, and related enzymatic modifications.^69-71^ Beyond their classical role in lipid emulsification, BAs serve as signaling molecules that modulate GLP-1 secretion by activating GPRs such as Takeda G protein-coupled receptor 5 (TGR5) or by regulating the nuclear receptor FXR. The GM critically shapes BA pool composition and transformation patterns, making it a central player in this regulatory axis.^69-76^

Multiple GM taxa modulate GLP-1 secretion via bile acid metabolism through three principal mechanisms. First, *Clostridium* species express 7α/β-hydroxysteroid dehydrogenases that convert primary BAs like CDCA into secondary BAs such as UDCA and ωMCA. Notably, *Clostridium bolteae*-derived UDCA has been shown to alter BA composition and enhance GLP-1 release.^16^ Second, *Bacteroides* species facilitate the conversion of primary BAs into secondary derivatives such as ωMCA and HCA, providing ligands for GLP-1–secreting L cells.^35^ *Lachnoclostridium* generates LCA from primary BAs via 7α-dehydroxylation; LCA acts as an FXR antagonist and thereby promotes GLP-1 secretion.^72^ Third, several bacterial genera—including *Enterococcus*, *Streptococcus*, *Bacteroides*, *Lactobacillus*, and *Lactobacillus reuteri*—express bile salt hydrolase (BSH), an enzyme that catalyzes the deconjugation of BAs. Increased BSH activity typically reduces the availability of FXR antagonists (e.g., tauro-*β*-muricholic acid [TβMCA]) and TGR5 agonizts (e.g., UDCA, tauroursodeoxycholic acid [TUDCA]), thus suppressing GLP-1 secretion. In contrast, diosgenin and *L. reuteri JCM 1112* can inhibit BSH activity, restore the levels of active BAs, and facilitate GLP-1 release.^70^^,^^71^^,^^77^^,^^78^ Additionally, *Akkermansia muciniphila* indirectly enhances BA-induced stimulation of L cells by strengthening intestinal barrier integrity and improving enterohepatic circulation efficiency.^78^

BAs regulate GLP-1 secretion predominantly through TGR5- and FXR-dependent pathways. In the TGR5 pathway, secondary BAs—including ωMCA, HCA, UDCA, DCA, and taurolithocholic acid (TLCA)—bind to basolateral TGR5 on L cells, activating the Gαs–cyclic adenosine monophosphate (cAMP)–cAMP response element-binding protein (CREB) signaling cascade. This elevates intracellular cAMP and calcium levels, promoting GLP-1 exocytosis.^32^^,^^35^^,^^73-75^^,^^79^ TGR5 knockout or BA sequestration abolishes this response.^69^^,^^74^ In the FXR pathway, FXR antagonists generated by GM—such as TβMCA, UDCA, and LCA—or reduced BA deconjugation due to BSH inhibition suppress FXR signaling. This relieves FXR-mediated repression of proglucagon gene (Gcg) transcription, restores glycolytic function by upregulating phosphofructokinase 1 (Pfk1) and enolase 1 (Eno1), increases ATP production, and enhances GLP-1 synthesis.^16^^,^^72^^,^^77^^,^^78^^,^^80^ Our group has demonstrated that UDCA and glycoursodeoxycholic acid (GUDCA) derived from *Clostridium bolteae* act as FXR antagonists, directly promoting Gcg transcription and stimulating the proliferation of Lgr5⁺ intestinal stem cells (ISCs), thereby increasing the number of L cells and enhancing GLP-1 secretory capacity.^16^ Furthermore, certain GM also modulate BA sensitivity by regulating ASBT-mediated BA reabsorption in the ileum.^69^^,^^79^

Emerging evidence indicates that the GM-derived metabolite trimethylamine *N*-oxide (TMAO) attenuates GLP-1 secretion. This suppression operates through two principal mechanisms: an indirect pathway involving bile acid-mediated signaling and a direct pathway linked to cellular stress. Specifically, TMAO downregulates the expression of cholesterol 7α-hydroxylase (Cyp7a1) and cytochrome P450 sterol 27-hydroxylase (Cyp27a1), key enzymes responsible for converting cholesterol into primary bile acids, thereby depleting the total bile acid pool.^81^^,^^82^ Under physiological conditions, BAs act as potent agonizts for TGR5 located on the basolateral surface of L cells. Activation of TGR5 stimulates intracellular cAMP signaling and promotes GLP-1 granule exocytosis.^32^^,^^83^ A TMAO-induced reduction in luminal bile acids thus attenuates TGR5 activation, resulting in diminished postprandial GLP-1 release and glucose intolerance. Independently, TMAO can directly impair L cell function by inducing endoplasmic reticulum (ER) stress. In professional secretory cells such as L cells and pancreatic *β*-cells, maintenance of ER homeostasis is essential for hormone production. At pathological concentrations, TMAO activates the PERK branch of the unfolded protein response and elevates ROS, collectively disrupting Gcg processing and GLP-1 maturation.^84-86^ Importantly, the effects of TMAO must be distinguished from those of its dietary precursors—such as choline and betaine—which can promote GLP-1 secretion via muscarinic or transporter-dependent mechanisms.^87^ Microbial conversion of these precursors to TMAO abolishes such benefits, whereas inhibition of TMA lyase with agents such as 3,3-dimethyl-1-butanol (DMB) restores bile acid synthesis and GLP-1 responsiveness.^88^

### 2.3 LPS-mediated GLP-1 regulation

LPS, a major component of the outer membrane of Gram-negative bacteria, is a potent pro-inflammatory endotoxin, with its lipid A moiety serving as the principal activator of innate immune responses. Under normal physiological conditions, LPS remains confined to the intestinal lumen due to the integrity of the gut epithelial barrier. However, when the barrier is compromised—due to inflammation, ischemia, or psychological stress—LPS can translocate and directly interact with intestinal L cells, significantly influencing GLP-1 secretion.^89^^,^^90^

Several Gram-negative bacterial genera are primary sources of LPS in the gut. *Escherichia coli* is the most well-characterized LPS-producing strain, and its intestinal overgrowth can markedly elevate luminal and systemic LPS levels.^89^^,^^90^ Other genera, including *Bacteroides* and *Proteus*, contribute to LPS release, particularly under conditions of dysbiosis, polymicrobial infection, or impaired epithelial integrity.^90^^,^^91^ An increased abundance of *Enterococcus* has also been associated with LPS-driven inflammatory responses.^92^ In contrast, commensal bacteria such as *Bifidobacterium* and *Ruminococcus* may exert protective effects by limiting the expansion of LPS-producing bacteria and enhancing gut barrier function, thereby indirectly reducing LPS translocation.^54^^,^^92^

The regulatory effects of LPS on GLP-1 are mediated through two interconnected mechanisms: direct receptor-dependent pathways and intestinal barrier-dependent modulation. First, LPS directly activates toll-like receptor 4 (TLR4) expressed on enteroendocrine L cells. This activation initiates intracellular calcium signaling cascades, which in turn rapidly upregulate the expression of Gcg and the prohormone convertase prohormone convertase 1/3 (PC1/3)—key molecules for GLP-1 biosynthesis. Consequently, this pathway promotes GLP-1 synthesis and secretion in a manner independent of early-stage changes in proinflammatory cytokines.^89^ Second, LPS impairs the intestinal epithelial barrier by disrupting tight junction proteins, including occludin, claudin-1, and zonula occludens-1 (ZO-1). This disruption increases epithelial permeability, thereby enhancing LPS penetration to the underlying L cells and amplifying LPS-mediated effects on GLP-1 regulation.^54^^,^^92^ Notably, functional redundancy exists in LPS-sensing receptors within this context. In TLR4-deficient models, alternative pattern recognition receptors—such as TLR2 and TLR7—can partially compensate for the loss of TLR4. These alternative receptors promote GLP-1 secretion via downstream proinflammatory cytokines, including interleukin-6 (IL-6) and TNF-α.^54^^,^^92^ Critical to this regulatory network is the expression of functional TLR4 on enteroendocrine L cells, as well as the pivotal role of intestinal epithelial barrier integrity in determining L cell responsiveness to LPS. Specifically, low-dose LPS induces GLP-1 secretion only when the intestinal barrier is compromised. This LPS-driven GLP-1 secretion depends on the activation of TLR4-dependent signaling, which triggers intracellular calcium flux in L cells to drive hormone release. Importantly, the secreted GLP-1 further exerts anti-inflammatory effects and contributes to the restoration of intestinal mucosal integrity—forming a feedback loop that modulates both inflammatory responses and barrier function.^92^

Current evidence indicates that the influence of bacterial endotoxins on enteroendocrine L cells exhibits a biphasic dose–response relationship, shaped primarily by the magnitude of inflammatory stimulation. Under conditions such as metabolic endotoxemia or early infection, low-dose LPS serves as an adaptive metabolic signal rather than a purely cytotoxic agent.^89^^,^^93^^,^^94^ As shown by Lebrun et al., intravenous administration of LPS (2 nanogram [ng]/kilogram [kg]) in healthy volunteers significantly increased plasma GLP-1 levels compared with saline controls.^89^ In contrast, high-dose LPS exposure,^95^ as seen in severe sepsis, shifts L cell function from adaptive to pathological. Although circulating GLP-1 may transiently increase due to stress-induced degranulation, sustained inflammatory load impairs L cell regenerative capacity. Gagnon et al. demonstrated that chronic exposure to TNF-α—a central mediator in high-grade endotoxemia—activates JNK signaling and suppresses Gcg transcription.^96^ Thus, LPS operates as a functional switch: at lower levels it promotes GLP-1 release to support metabolic homeostasis, whereas at cytotoxic concentrations it drives L cell failure and GLP-1 resistance.

### 2.4 Tryptophan metabolite-mediated GLP-1 regulation

Tryptophan metabolites regulate the secretion of GLP-1 and are primarily produced via two pathways. One is the host’s intrinsic metabolic pathways, including the serotonin pathway (which converts tryptophan into serotonin, or 5-HT) and the kynurenine pathway (which generates kynurenine). The other is the genetically modified microbiota-dependent pathway: these GM microbes convert tryptophan into indole derivatives such as indole, indole-3-lactic acid (ILA), indole-3-propionic acid (IPA), and indole-3-acetic acid (IAA).

Certain *Lactobacillus* species, such as *L. salivarius*, *L. animalis*, and *L. delbrueckii*, produce ILA, which promotes the differentiation of intestinal stem cells into GLP-1–secreting L cells by upregulating transcription factors including Math1, Neurog3, and Neurod1, while concurrently suppressing Notch signaling.^97^ Indole-producing bacteria such as *Escherichia coli* and *Clostridium* directly stimulate colonic L cells to release GLP-1 and activate vagal signaling through the GLP-1R in the central nervous system.^98^ Pharmacological interventions also impact this pathway; for instance, dapagliflozin has been shown to enrich E. coli and Clostridium populations, thereby increasing intestinal tryptophan and IAA levels. These metabolites, in turn, promote GLP-1 synthesis by upregulating Gcg and prohormone PC1/3 expression via activation of GPR142 on L cells.^99^ Different bacteria produce distinct tryptophan metabolites due to the presence of specific key enzymes that catalyze these pathways. Other GM members, including *Akkermansia muciniphila, Lachnospiraceae* NK4A136 group, and *Tannerellaceae*, metabolize tryptophan predominantly through the indole pathway. Their derivatives enhance GLP-1 secretion by activating the aryl hydrocarbon receptor (AhR) and improving gut barrier integrity.^100^ Notably, treatment with liraglutide has been associated with elevated colonic levels of IPA, which may indirectly support GLP-1 secretion by modulating the local metabolic microenvironment.^101^

GM, through its extensive metabolite network, functions as a central upstream regulator of GLP-1 secretion. Key microbial metabolites—including SCFAs, BAs, LPS, and tryptophan derivatives—modulate L cell activity through specific signaling cascades. These include membrane-bound receptors such as FFAR2/3, TGR5, TLR4, and the AhR; nuclear transcription factors such as FXR; and intracellular mediators such as cAMP and Ca²⁺. In parallel, microbial metabolites contribute to the stabilization of the intestinal microenvironment by enhancing barrier integrity and suppressing inflammation, collectively fine-tuning GLP-1 synthesis and secretion.

Recent findings indicate that inter-individual differences in GM composition can meaningfully influence GLP-1 secretory responses. Notably, stronger GLP-1 induction has been observed in individuals whose microbiota harbor greater fiber-fermenting and SCFAs-producing capacity.^102^ Rather than overall diversity metrics, microbial functional outputs—such as SCFAs, BAs, and indole derivatives—appear to be the primary drivers of GLP-1 regulation through GPCR-, TGR5-, and AhR-mediated pathways. These observations underscore that the metabolic potential of the GM, rather than its taxonomic diversity alone, is more relevant for predicting host GLP-1 responsiveness.^36^^,^^103^

These pathways form an integrated regulatory network. SCFAs and beneficial secondary BAs serve as “metabolic enhancers,” directly promoting GLP-1 release. Select tryptophan metabolites act as “differentiation cues,” supporting L cell lineage development and epithelial repair. In contrast, LPS operates as a “stress signal,” triggering inflammatory responses and GLP-1 dysregulation when the epithelial barrier is compromised. In the context of degenerative musculoskeletal diseases, GM dysbiosis—characterized by reduced SCFAs production, depletion of beneficial BAs, and increased LPS translocation—is likely to impair GLP-1 signaling, thereby contributing to bone loss, muscle atrophy, and systemic metabolic imbalance.

Targeting the GM–metabolite–L cell–GLP-1 axis holds considerable therapeutic potential. Emerging strategies include dietary interventions (e.g., fiber or tryptophan enrichment), administration of tailored probiotics or prebiotics, FMT, and the development of selective receptor agonizts (e.g., TGR5, FFAR2) or FXR antagonists. These approaches aim to restore host–microbiota metabolic dialog, enhance endogenous GLP-1 production, and ultimately mitigate the progression of degenerative musculoskeletal diseases.

## 3. Mechanisms of GLP-1 action in degenerative musculoskeletal diseases

### 3.1 GLP-1 as a cross-tissue regulatory factor

#### 3.1.1 Distribution of GLP-1R

GLP-1R is well-established for its regulatory roles in glycemic control and appetite modulation, primarily through its expression in the pancreas, gastrointestinal tract, and central nervous system. In recent years, accumulating evidence has demonstrated the expression of GLP-1R across a wide range of musculoskeletal tissues, providing a robust molecular basis for its involvement in degenerative musculoskeletal diseases. In OA, GLP-1R expression shows marked tissue and cellular specificity, with a conserved distribution pattern observed across species including humans, rats, and mice. Chondrocytes are a principal site of GLP-1R expression, with the receptor predominantly localized to the cell membrane in both healthy and degenerated cartilage. This has been confirmed via immunohistochemistry, Western blotting, and immunofluorescence in rat,^104^^,^^105^ human,^41^^,^^106^ and mouse^16^^,^^41^ knee cartilage. For example, Chen et al. identified GLP-1R in both normal rat chondrocytes and cultured cartilage cells,^104^ while Huang et al. verified its expression in the human C28/I2 chondrocyte cell line.^106^ Beyond cartilage, synovial tissue also exhibits significant GLP-1R expression. In human OA synovium, GLP-1R-positive cells are enriched in the intimal layer and perivascular regions,^41^ whereas in mouse synovium, expression is localized to synovial cell clusters.^16^^,^^41^ Notably, Yang et al. confirmed that gut-derived GLP-1 can activate synovial GLP-1R to exert chondroprotective effects.^16^ Furthermore, in mouse knees, GLP-1R is also detected in the meniscus and bone marrow cells,^16^^,^^41^ and even on the membrane and intracellularly in RAW264.7 macrophages *in vitro*.^41^ These findings suggest that GLP-1R has a broader role beyond cartilage, potentially contributing to the regulation of OA-associated inflammation and disease progression. In OP, GLP-1R is broadly expressed in multiple cell types involved in bone remodeling. Osteocytes, the most abundant and metabolically active bone cells, express GLP-1R in both in vivo models and in cell lines such as IDG-SW3.^39^^,^^107^ Bone marrow-derived macrophages (BMSCs), crucial osteoprogenitors, are also confirmed to express GLP-1R at high levels.^39^^,^^108-110^ Likewise, the MC3T3-E1 osteoblast precursor line and primary osteoblasts both show robust GLP-1R expression.^109-113^ Additionally, osteoclasts and their precursors also express GLP-1R, highlighting its regulatory involvement across both anabolic and catabolic arms of bone metabolism.^39^^,^^114^^,^^115^ These findings provide a comprehensive cellular framework supporting the role of GLP-1 in modulating skeletal homeostasis. In SP, the expression and functional significance of GLP-1R in skeletal muscle cells have been well validated across various physiological and pathological states, both *in vitro* and in vivo. Under normal conditions, GLP-1R is expressed in skeletal muscles such as the gastrocnemius, tibialis anterior, and soleus of C57BL/6J mice. Co-immunoprecipitation assays demonstrate direct binding between GLP-1 and GLP-1R, and the receptor remains detectable in differentiated C2C12 myotubes.^40^ Genetic validation using GLP-1R^flox/flox^ mice crossed with *β*-actin-Cre mice showed a 50% reduction of GLP-1R expression in heterozygotes and near-complete loss in homozygotes, further confirming its endogenous expression in skeletal muscle tissue.^116^ Importantly, in pathological contexts such as SP mouse models or obesity-induced muscle dysfunction, GLP-1R expression is maintained rather than diminished, suggesting it remains a viable therapeutic target.^17^^,^^117^^,^^118^ In diabetes-related muscle injury models—such as streptozotocin (STZ)-induced diabetic rats, db/db diabetic mice, and high glucose-treated C2C12 myotubes—GLP-1R expression in skeletal muscle remains stable.^119–121^ Additionally, in disuse atrophy models and inflammatory myopathies, GLP-1R is highly expressed in the sarcolemma of inflamed fibers, cytoplasm of apoptotic fibers, and muscle satellite cells.^122^ Stable GLP-1R expression has also been observed in muscle tissues from chronic kidney disease-related muscle wasting and Duchenne muscular dystrophy (DBA/2J-mdx) models,^116^ underscoring its consistent presence across diverse disease states. In IVDD, GLP-1R expression is detected in various disc-associated tissues. Immunohistochemical and immunofluorescence staining reveal the presence of GLP-1R-positive cells in both healthy and degenerated human disc tissue, indicating preserved expression across disease stages. Similarly, significant GLP-1R expression has been confirmed in rat intervertebral disks, suggesting evolutionary conservation.^123^ At the cellular level, nucleus pulposus cells (NPCs) are primary sites of GLP-1R expression. Compared to 293 T cells with low endogenous expression, NPCs display markedly higher GLP-1R protein levels via Western blot, further confirmed through plasmid overexpression studies.^123^ In both human and rat NPCs, GLP-1R protein (~53 kDa) is consistently detectable. Although GLP-1R expression may be downregulated during degeneration, it remains functionally present, supporting its role in disc homeostasis and repair.

It is noteworthy that GLP-1R expression across musculoskeletal tissues exhibits pathological sensitivity—often downregulated under conditions of degeneration, inflammation, or metabolic stress. However, GLP-1 or its agonizts can reactivate GLP-1R expression or enhance its signaling function under these conditions. This dual role—acting as both a stress sensor and a homeostatic regulator—underscores the therapeutic promise of GLP-1R as a systemic intervention target in degenerative musculoskeletal disorders. Its widespread, dynamic, and responsive expression profile offers a compelling histological foundation for targeted therapies aimed at restoring tissue integrity and metabolic balance.

#### 3.1.2 Typical mechanism modules and functional networks

**Figure 3.**
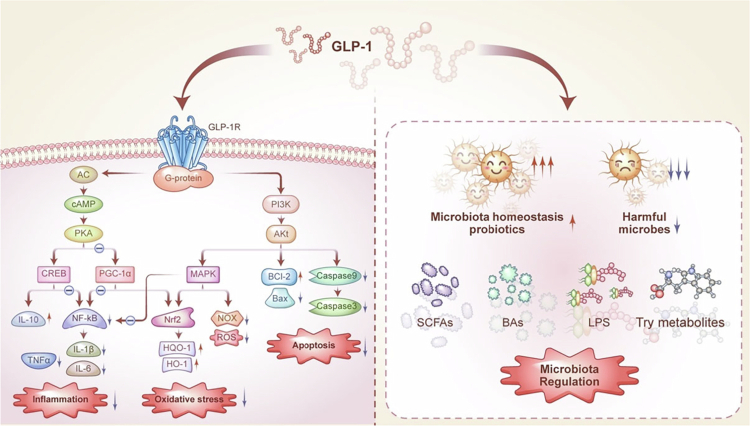
GLP-1 exerts its biological effects through two primary mechanisms. On one hand, it activates the GLP-1R via G protein-coupled signaling, thereby regulating three key cellular processes: inflammation, oxidative stress, and apoptosis. GLP-1 alleviates inflammation through two key mechanisms: first, it downregulates the expression of pro-inflammatory cytokines (including TNF-*α*, IL-1β, and IL-6) by leveraging the CREB, PGC-1α, and MAPK pathways to inhibit the NF-κB; second, it simultaneously upregulates the anti-inflammatory cytokine IL-10 via the CREB pathway. This dual modulation of pro-inflammatory and anti-inflammatory factors effectively attenuates inflammatory responses. In terms of oxidative stress, GLP-1 signaling activates the MAPK pathway and PGC-1α, which subsequently enhances the activity of nuclear factor erythroid 2-related factor 2 (Nrf2)—a pivotal transcription factor for antioxidant defense. This leads to increased expression of antioxidant enzymes such as HO-1 and NQO1, while decreasing the production of ROS and NOX, thereby reducing oxidative damage. GLP-1 also mitigates apoptosis by activating the phosphatidylinositol 3-kinase-protein kinase B (PI3K–Akt) signaling pathway. This results in elevated expression of the anti-apoptotic protein BCL-2 and reduced levels of pro-apoptotic proteins including Bax, Caspase-9, and Caspase-3, effectively inhibiting cell apoptosis. On the other hand, GLP-1 plays a vital role in preserving gut microbial balance by enhancing the growth of commensal bacteria and inhibiting the expansion of detrimental species. Furthermore, GLP-1 signaling influences microbial-derived metabolites, such as SCFAs, BAs, LPS, underscoring a dynamic bidirectional crosstalk between host GLP-1 activity and GM metabolism. GLP-1: glucagon-like peptide-1; GLP-1R: GLP-1 receptor; TNF-α: tumor necrosis factor-alpha; IL-1β: interleukin-1 beta; IL-6: interleukin-6; CREB: cAMP response element-binding protein; PGC-1α: peroxisome proliferator-activated receptor gamma coactivator 1-alpha; MAPK: mitogen-activated protein kinase; NF-κB: nuclear factor kappa B; IL-10: interleukin-10; Nrf2: nuclear factor erythroid 2-related factor 2; HO-1: heme oxygenase-1; NQO1: NAD(*P*)H:quinone oxidoreductase 1; ROS: reactive oxygen species; NOX: NADPH oxidase; PI3K: phosphatidylinositol 3-kinase; BCL-2: B-cell lymphoma 2; Bax: B-cell lymphoma 2–associated X protein; SCFAs: short-chain fatty acids; BAs: bile acids; LPS: lipopolysaccharides; GM: gut microbiota.

Despite disease variations, GLP-1's actions converge on four core functional modules: anti-inflammation, antioxidant stress, anti-apoptosis, and microbiota regulation (Figure 3).

##### 3.1.2.1 Anti-inflammatory module.

Chronic inflammation is a fundamental pathological driver of degenerative musculoskeletal diseases, contributing to synovitis, bone marrow microenvironmental imbalance, inflammatory metabolic dysfunction in skeletal muscle, and IVDD. GLP-1 regulates immune polarization, cytokine production, and inflammatory gene transcription through multiple interconnected pathways, thereby forming a multilayered anti-inflammatory network. One core mechanism involves the suppression of NF-κB signaling pathway. Upon binding to the GLP-1R, GLP-1 reduces the phosphorylation of IκBα, thereby preventing the nuclear translocation of NF-κB p65 and downregulating the expression of pro-inflammatory cytokines such as TNF-*α*, IL-6, and IL-1β.^41^^,^^124^^,^^125^ This mechanism has been consistently validated across multiple disease models, including synovial macrophages in OA,^41^ bone marrow precursor cells in OP,^124^ and muscle cells in SP.^126^ In parallel, GLP-1 activates adenylate cyclase (AC), leading to increased intracellular cAMP levels, which then activate protein kinase A (PKA). PKA subsequently phosphorylates CREB, enhancing its transcriptional activity. CREB not only promotes anti-inflammatory cytokine interleukin-10 (IL-10) expression but also inhibits NF-κB signaling, thereby further reducing pro-inflammatory cytokine production.^41^^,^^42^ Additionally, GLP-1 activates peroxisome proliferator-activated receptor gamma coactivator 1-alpha (PGC-1α) via the cAMP–PKA axis, which stabilizes mitochondrial function and attenuates inflammatory mediator release. Moreover, GLP-1 signaling through the PI3K–Akt pathway also activates MAPK, contributing further to NF-κB inhibition.

In summary, GLP-1 orchestrates a robust anti-inflammatory program through three core axes—PKA–CREB, PGC-1α, and MAPK—that converge on NF-κB suppression. This integrated signaling framework supports both the inhibition of pro-inflammatory mediators and the promotion of anti-inflammatory repair, demonstrating conserved functionality across OA, SP, OP, and IVDD. This anti-inflammatory module forms a key foundation of GLP-1's tissue-protective effects in degenerative musculoskeletal diseases.

##### 3.1.2.2 Anti-oxidant stress regulation module.

GLP-1 also exerts potent antioxidant effects through the modulation of multiple intracellular signaling pathways, regulating cellular metabolism, antioxidant enzyme expression, and redox homeostasis. Following GLP-1–GLP-1R binding, the AC–cAMP–PKA axis upregulates PGC-1α, which not only suppresses inflammation via NF-κB inhibition but also plays a central role in mitochondrial bioenergetics, enhancing fatty acid oxidation and improving antioxidant capacity. PGC-1α activation promotes the transcriptional activity of Nrf2,^117^^,^^127^ a master regulator of antioxidant defense. Nrf2 subsequently upregulates antioxidant genes such as heme oxygenase-1 (HO-1) and NAD(*P*)H:quinone oxidoreductase 1 (NQO1), which reduce oxidative stress and mitigate cellular injury. Simultaneously, GLP-1 signaling through the PI3K–AKT–MAPK axis enhances Nrf2 nuclear translocation, increasing cellular resilience to oxidative insult.^128^ Furthermore, MAPK signaling downregulates NADPH oxidase (NOX) activity, reducing the generation of ROS.^111^^,^^124^

Through the coordinated action of these pathways, GLP-1 establishes a comprehensive antioxidant defense network—from redox stabilization and mitochondrial protection to ROS clearance. These mechanisms have been demonstrated in various degenerative musculoskeletal disease models, including OA, OP, SP, and IVDD, supporting the broad cytoprotective role of GLP-1 against oxidative damage.

##### 3.1.2.3 Anti-apoptosis regulation module.

GLP-1 inhibits apoptosis primarily through activation of the PI3K–AKT signaling pathway following GLP-1R engagement. Activated AKT suppresses pro-apoptotic Bcl-2–associated X protein (Bax) expression,^42^^,^^104^ thereby preventing mitochondrial outer membrane permeabilization and the release of cytochrome c, which initiates caspase-dependent apoptosis. Concurrently, AKT enhances the expression of the anti-apoptotic protein Bcl-2, maintaining mitochondrial membrane integrity and further inhibiting apoptotic signaling.^111^^,^^129^^,^^130^ Moreover, AKT suppresses downstream apoptotic effectors, including Caspase-9 and Caspase-3.^131^ Caspase-9, an initiator caspase, activates Caspase-3, which cleaves key intracellular substrates to execute the apoptotic program. By inhibiting Caspase-9, GLP-1 indirectly downregulates Caspase-3 activation, attenuating apoptosis.

This anti-apoptotic effect has been validated in multiple degenerative musculoskeletal disease contexts. In OA, GLP-1 protects articular chondrocytes from apoptosis, preserving cartilage structure and function. In OP, GLP-1 supports osteoblast survival, reducing bone loss and improving bone regeneration. In SP, it prevents muscle cell apoptosis, supporting muscle mass and function. In IVDD, GLP-1 protects disc cells and promotes extracellular matrix synthesis, contributing to disc stability. Through inhibition of Bax, activation of Bcl-2, and suppression of Caspase-9/3 activity, GLP-1 forms a comprehensive survival-promoting network that preserves tissue integrity under degenerative stress.

##### 3.1.2.4 Microbiota regulation module.

Emerging evidence suggests that GLP-1 not only acts downstream of GM-derived metabolic signaling but also reciprocally regulates GM composition and function, forming a bidirectional regulatory loop with therapeutic relevance for degenerative musculoskeletal diseases. GLP-1 improves gut barrier integrity, modulates bile acid metabolism, and reduces inflammatory tone—establishing a favorable niche for beneficial bacteria and supporting the production of protective metabolites, including SCFAs, specific BAs, and indole derivatives. These metabolites, in turn, enhance anti-inflammatory responses, support bone metabolism, and promote muscle protein synthesis.

For instance, treatment with GLP-1 analogs such as liraglutide elevates colonic levels of IPA, thereby improving intestinal homeostasis and indirectly supporting GLP-1 production. GLP-1 also enhances tight junction protein expression, reducing LPS translocation and reshaping luminal conditions to suppress dysbiotic expansion. Additionally, by modulating bile acid metabolism, GLP-1 influences the abundance of BAs–transforming species such as *Clostridium bolteae*.^16^ SCFAs levels also rise indirectly in response to improved barrier function and microbial composition.^51^ Through its anti-inflammatory and barrier-protective actions, GLP-1 may also suppress LPS-producing Gram-negative bacteria.^91^

GLP-1 analogs have been shown to increase the abundance of SCFAs-producing genera such as *Roseburia* and *Ruminococcus*, and promote colonization of beneficial taxa like *Akkermansia muciniphila*. At the same time, they inhibit potential pathobionts including *Escherichia coli* and *Enterococcus* by reducing endotoxemia and restoring barrier function. This ecological shift—favoring beneficial over harmful microbes—amplifies the production of SCFAs and IPA, further reinforcing GLP-1’s anti-inflammatory, osteoanabolic, and myoprotective effects.

Thus, targeting the GLP-1 pathway not only offers direct systemic benefits but also enables the remodeling of the GM–metabolite axis, establishing a multimodal therapeutic strategy for the treatment of degenerative musculoskeletal diseases. The therapeutic promise of GLP-1 arises from its stable cross-tissue receptor expression, high responsiveness under stress, and the integrated functionality of its anti-inflammatory, antioxidant, anti-apoptotic, and microbiota-regulating modules. Collectively, GLP-1 represents a central node in the coordinated regulation of metabolic inflammation, tissue degeneration, and musculoskeletal homeostasis.

### 3.2 Disease-specific roles of GLP-1 in degenerative musculoskeletal diseases

At the level of shared mechanisms, GLP-1 exerts a range of protective effects across bone- and muscle-related tissues through GLP-1R, including anti-inflammatory activity, suppression of oxidative stress, inhibition of apoptosis, and modulation of GM composition. Together, these actions form a stable and multifunctional tissue-protective network. However, the effects of GLP-1 are not uniform across all degenerative musculoskeletal diseases; rather, its disease-specific efficacy is closely aligned with the predominant pathological drivers in each condition. In OA, the core pathological loop involves the progression from synovitis to cartilage degradation. OP is characterized by a metabolic imbalance between decreased osteogenesis and increased osteoclastogenesis. SP is primarily driven by impaired myogenesis and progressive muscle atrophy. IVDD is dominated by NPCs injury and extracellular matrix (ECM) breakdown. GLP-1 demonstrates disease-targeted therapeutic advantages by modulating local immune and metabolic microenvironments. It exerts distinct effects in each disease context: anti-inflammatory and chondroprotective actions in OA; stimulation of osteoblast differentiation and inhibition of osteoclast activity in OP; promotion of myogenesis and attenuation of muscle wasting in SP; and cytoprotection and ECM preservation in IVDD. These effects have been increasingly validated through both preclinical and clinical studies. To further elucidate the clinical potential of GLP-1–based therapies, it is essential to examine its specific roles and mechanistic evidence in relation to the unique pathological characteristics of each degenerative musculoskeletal disease (Table 1).

**Table 1.** Specific Mechanisms of GLP-1 in Degenerative Musculoskeletal Diseases.

Disease	Pathological Core	GLP-1 Mechanism of Action	Primary Targets	Research Evidence
OA	Cartilage matrix degradation Synovitis	Promotes matrix synthesis (COL2, ACAN) Inhibits matrix degradation (MMPs, ADAMTS) Reduces synovitis (polarization of synovial macrophages)	Chondrocytes Synovial macrophages	*In vitro*: Human/mouse chondrocytes Animal models: ACLT/MIA/DMM OA models Clinical: STEP 9 trial
OP	Reduced osteogenesis Enhanced osteoblastogenesis	Promotes osteogenesis (RUNX2, ALP, OC) Inhibits osteoclastogenesis (OPG/RANKL)	Osteoblasts (MC3T3-E1, BMSCs) Osteoclasts (RAW264.7)	*In vitro*: Osteoblasts/osteoclasts Animal models: OVX/diabetic OP models Clinical: Multi-national cohorts
SP	Impaired myogenesis Accelerated atrophy	Promotes myogenesis (MyoD, MyoG) Inhibits atrophy factors (Atrogin-1, MuRF-1) Improves mitochondrial function	Myoblasts (C2C12) Mitochondrial metabolic pathways in muscle fibers	*In vitro*: C2C12 model Animal models: Diabetic/disuse-induced atrophy SP models Clinical: Cohort studies
IVDD	Nucleus pulposus cell injury ECM disruption	Inhibits abnormal NPCs death Upregulates ECM synthesis factors (ACAN, COL2)	Nucleus pulposus cells (NPCs) ECM components	*In vitro*: Human/rat NPCs Animal models: Caudal puncture IVDD models No clinical studies available

OA: Osteoarthritis, OP: Osteoporosis, SP: Sarcopenia, IVDD: Intervertebral Disc Degeneration, ECM: Extracellular Matrix, COL2: Collagen Type II, ACAN: Aggrecan, MMPs: Matrix Metalloproteinases, ADAMTS: A Disintegrin and Metalloproteinase with Thrombospondin Motifs, RUNX2: Runt-related transcription factor 2, ALP: Alkaline Phosphatase, OC: Osteocalcin, OPG: Osteoprotegerin, RANKL: Receptor Activator of Nuclear Factor κB Ligand, MyoD: Myogenic Differentiation 1, MyoG: Myogenin, Atrogin-1: Muscle Atrophy F-box protein 1, MuRF-1: Muscle RING-finger protein-1, NPCs: Nucleus Pulposus Cells, MC3T3-E1: Mouse Calvaria 3T3 E1 Cell Line, BMSCs: Bone Marrow-derived Mesenchymal Stem Cells, ACLT: Anterior Cruciate Ligament Transection, MIA: Monosodium Iodoacetate, DMM: Destabilization of the Medial Meniscus, STEP 9: Semaglutide Treatment Effect in People with Obesity 9, OVX: Ovariectomy.

Across musculoskeletal degenerative diseases, preclinical evidence consistently identifies the GM–GLP-1 axis as a significant regulatory pathway. Cellular studies indicate that microbial metabolites, including SCFAs and BAs, stimulate GLP-1 secretion and trigger several protective responses in target cells.^16^^,^^103^^,^^132^ These responsive mechanisms involve NF-κB inhibition in chondrocytes,^133^ osteogenic differentiation through cAMP/PKA–β-catenin in osteoblasts,^129^ suppression of osteoclastogenesis via NFATc1 downregulation,^134^ enhanced mitochondrial biogenesis through AMPK–PGC-1α in myocytes,^135^ and reduced apoptosis and dysregulated autophagy in NPCs via PI3K/Akt/mTOR signaling.^128^ Animal studies further support these findings, showing that modulation of the GM–GLP-1 axis helps alleviate structural degeneration and functional impairment. For example, in models of OA and OP, GLP-1 receptor agonizts or microbial interventions have been found to reduce cartilage loss, improve bone microstructure, and rebalance osteoprotegerin (OPG)/receptor activator of nuclear factor kappa-B ligand (RANKL) signaling.^16^^,^^136^^,^^137^ Similarly, in IVDD models, GLP-1-based approaches help preserve NPCs and delay disc height loss.^123^ In the context of SP, GLP-1 signaling appears to enhance glucose utilization and support muscle mass in animal models.^138^^,^^139^ However, human studies suggest that GLP-1-based therapies—widely used for weight loss—may lead to a reduction in lean body mass due to substantial caloric and protein restriction. Therefore, their potential application in SP requires careful consideration, with adjunct strategies such as high-protein diets and resistance training recommended to mitigate muscle loss.^140-143^ Together, these cellular and animal studies provide complementary insights, positioning the GM–GLP-1 axis as an integrative modulator of musculoskeletal homeostasis.

#### 3.2.1 Specific role of GLP-1 in OA

Unlike other degenerative musculoskeletal disorders, the pathological hallmark of OA lies in a vicious cycle between synovitis and cartilage matrix degradation. The therapeutic efficacy of GLP-1 in OA therefore relies heavily on its dual ability to regulate synovial macrophage polarization and to potently inhibit cartilage matrix-degrading enzymes. This dual mechanism underpins its unique therapeutic advantage in OA. Specifically, macrophage modulation ameliorates the inflammatory microenvironment within the synovium, while inhibition of matrix-degrading enzymes directly prevents cartilage degeneration. Together, these actions precisely target the central pathological axis of OA. Distinct from its roles in other metabolic disorders, GLP-1 in OA exerts its effects primarily through the coordinated regulation of local immune responses and cartilage homeostasis. On the one hand, it modulates the immunological status of synovial tissue; on the other, it promotes type II collagen (COL2) and aggrecan (ACAN) synthesis, reduces glycosaminoglycan (GAG) release, and maintains the structural and functional integrity of cartilage. This dual strategy—anti-inflammatory action combined with cartilage protection—aligns closely with the core pathological feature of OA, namely “synovitis-induced cartilage degradation,” and thus distinguishes GLP-1 from therapeutic approaches used in other degenerative musculoskeletal diseases.

The mechanisms and efficacy of GLP-1 and GLP-1R agonizts (GLP-1RAs) in OA have been progressively elucidated through *in vitro*, in vivo, and clinical studies. In cellular models, liraglutide has demonstrated pronounced chondroprotective effects. *In vitro* experiments using IL-1β-stimulated rat chondrocytes revealed that liraglutide suppresses the expression of matrix metalloproteinases MMP-3 and matrix metalloproteinase 13 (MMP-13), as well as a disintegrin and metalloproteinase with thrombospondin motifs 5 (ADAMTS-5), while enhancing COL2 expression—effects that are reversed by GLP-1 receptor knockdown.^104^ In human primary chondrocytes, liraglutide dose-dependently inhibited TNF-*α*-induced MMP-3/13 and ADAMTS-4/5 expression, while restoring the expression of COL2 and ACAN.^42^ Similar protective effects were observed in mouse primary chondrocytes, where liraglutide reduced IL-1β-induced GAG release, downregulated the expression of the ER stress marker C/EBP homologous protein (CHOP), and promoted macrophage polarization from the pro-inflammatory M1 to the anti-inflammatory M2 phenotype.^41^

Animal studies further validate the therapeutic potential of GLP-1 pathway activators in various OA models. In a rat anterior cruciate ligament transection (ACLT) model, 8-week liraglutide treatment significantly reduced osteophyte formation, cartilage erosion, and the OA Research Society International (OARSI) score.^104^ In a mouse monoiodoacetate (MIA)-induced OA model, intra-articular liraglutide administration alleviated synovitis, elevated mechanical withdrawal thresholds, and demonstrated superior analgesic effects compared to dexamethasone.^41^ In a destabilization of the medial meniscus (DMM) model, the GLP-1 pathway agonist GUDCA decreased osteophyte formation and pain, and reduced the visual analog scale (VAS) score—effects that were reversed by the GLP-1 receptor antagonist Exendin 9-39.^16^ In a separate MIA-induced rat OA model, the GLP-1R agonist geniposide (GEN) activated autophagy via the GLP-1R/AMPK/mTOR axis, suppressed MMP-13, and upregulated COL2; again, these effects were blocked by Exendin 9-39.^106^

Population-based studies offer additional multi-dimensional evidence. The Shanghai OA Cohort Study, comprising 1807 knee OA patients with type 2 diabetes mellitus, revealed that GLP-1RAs users exhibited greater weight loss, slower medial tibiofemoral cartilage loss, a reduced incidence of knee surgery, and improved Western Ontario and McMaster Universities Osteoarthritis Index (WOMAC) pain scores compared to non-users. Mediation analysis indicated that weight loss partially accounted for the reduced surgical risk.^144^ Another cohort study (*n*  =  5972) demonstrated that OA patients using UDCA (a GLP-1 pathway activator) had a significantly reduced risk of knee replacement. Among OA patients with concurrent diabetes, GLP-1RAs users had lower joint replacement rates than non-users.^16^ The semaglutide treatment effect in people with obesity 9 (STEP 9) clinical trial (*n*  =  407) further confirmed that weekly subcutaneous administration of 2.4 milligrams (mg) semaglutide over 68 weeks led to significantly greater weight loss and larger reductions in WOMAC pain than placebo, alongside a reduction in analgesic medication use (including an non-steroidal anti-inflammatory drugs[NSAIDs]-sparing effect).^145^

Collectively, evidence from cellular, animal, and clinical research supports the effectiveness and mechanistic uniqueness of the GLP-1 signaling pathway as a therapeutic target for OA. In particular, among OA patients with comorbid obesity or diabetes, GLP-1's combined benefits in metabolic regulation and local lesion mitigation offer significant clinical promise. Future investigations should explore differential responses among OA subtypes, long-term safety profiles of GLP-1RAs, and potential combinatorial therapies (e.g., with GM modulation) to pave the way for their integration into routine clinical management of OA.

#### 3.2.2 Specific role of GLP-1 in OP

Unlike other degenerative musculoskeletal diseases, the pathological core of OP lies in dysregulated bone metabolism, characterized by a vicious cycle of reduced osteogenic activity and heightened osteoclastic resorption. Thus, the protective effect of GLP-1 in OP primarily relies on its bidirectional regulation of key bone-metabolizing cells and its inhibitory action on osteoclastogenesis. This dual mechanism grants GLP-1 a distinctive advantage in OP intervention. Specifically, GLP-1 enhances bone mass and architecture by promoting osteoblast proliferation, differentiation, and mineralization, while simultaneously suppressing bone resorption by inhibiting the differentiation of osteoclast precursors and modulating the local immune microenvironment. These effects directly address the underlying metabolic imbalance of OP and distinguish GLP-1 as a unique therapeutic candidate. Beyond restoring skeletal homeostasis, GLP-1 also mitigates diabetes-related bone loss, further highlighting its clinical potential in the management of OP.

At the cellular level, GLP-1R expression has been consistently identified on multiple bone-related cell types, including osteoblast precursors (e.g., MC3T3-E1), BMSCs, mature osteoblasts, and osteoclast precursor cells (e.g., BMDMs, RAW264.7). This provides a molecular basis for the direct action of GLP-1 and its analogs on bone metabolism.^39^^,^^109^^,^^111-114^^,^^125^ Functionally, GLP-1 exerts dual regulatory effects on bone metabolism. In osteogenic cells, GLP-1 promotes proliferation (e.g., 100 nanomolar (nM) liraglutide induced peak proliferation in MC3T3-E1 cells at 48 hours), migration, and osteogenic differentiation, as evidenced by the upregulation of RUNX2, OPN, alkaline phosphatase (ALP), osteocalcin (OC), and procollagen type I *N*-terminal propeptide (PINP), enhanced ALP activity, and increased mineralization capacity. Furthermore, GLP-1 mitigates apoptosis induced by external insults such as dexamethasone, cholesterol, and advanced glycation end-products (AGEs), and improves autophagic responses via the miR-27a-3p/GLP-1R axis.^110^^,^^129^^,^^146-151^ In osteoclast precursors, GLP-1 suppresses differentiation and resorptive activity by inhibiting the NF-κB/MAPK pathway and modulating macrophage polarization from M1 (pro-inflammatory) to M2 (anti-inflammatory) phenotypes.^148^^,^^149^^,^^151^ These effects are mediated by GLP-1R-dependent signaling pathways, including GLP-1R/ATP-binding cassette transporter A1 (ABCA1), GLP-1R/ERK, Wnt/LRP5/β-catenin (involving *β*-catenin Ser675 phosphorylation and nuclear translocation), and the Notch and Hedgehog cascades. Blockade of GLP-1R using Exendin 9-39 or inhibition of downstream effectors (e.g., ABCA1, *β*-catenin) abrogates these protective effects.^109^^,^^111^^,^^148^^,^^152^

In diverse OP models—such as ovariectomy (OVX), dexamethasone (DEX) induction, high-fat diet exposure, GLP-1R knockout, STZ/zucker diabetic fatty (ZDF)-induced diabetes, and ApoE^⁻/⁻^ dyslipidemia—GLP-1 administration has demonstrated consistent bone-protective effects. Morphologically, GLP-1 improves bone microarchitecture by significantly increasing bone mineral density (BMD), bone volume fraction (BV/TV), trabecular number (Tb.N), and trabecular thickness (Tb.Th), while reducing trabecular separation (Tb.Sp). These changes contribute to enhanced bone strength, reflected by higher femoral load-bearing capacity and stiffness, and reduced fracture risk. Notably, higher doses or prolonged treatment (e.g., 300 microgram [μg]/kg semaglutide weekly for 10 weeks) yield greater benefits.^107^^,^^130^^,^^153-160^ Metabolically, GLP-1 modulates bone turnover in a bidirectional manner: stimulating bone formation (elevating serum ALP, OC, PINP, and calcitonin levels; reducing sclerostin), while inhibiting bone resorption (lowering serum tartrate-resistant acid phosphatase [TRACP] and C-terminal telopeptide of type I collagen [CTX-I] levels; reducing TRAP-positive osteoclast numbers). GLP-1 promotes OPG expression and suppresses RANKL, thereby decreasing the RANKL/OPG ratio. Additionally, GLP-1 indirectly inhibits osteoclast activity via stimulation of calcitonin release from thyroid C-cells, a mechanism validated in GLP-1R-deficient mice.^147^^,^^148^^,^^150^^,^^152^^,^^160^^,^^161^ GLP-1 also alleviates bone damage associated with metabolic disturbances, such as dyslipidemia and hyperglycemia (e.g., lowering serum AGEs and improving lipid profiles). Combined treatment with liraglutide and insulin outperformed monotherapy in diabetic OP models. GLP-1R knockout mice exhibited increased osteoclastogenesis, reduced cortical bone mass, and heightened fracture susceptibility, reinforcing the critical role of GLP-1R in bone homeostasis.^148^^,^^150^^,^^162^

Emerging evidence indicates that the osteoprotective properties of GLP-1 extend beyond its systemic glycemic effects and involve direct metabolic reprogramming of bone cells. Given that bone remodeling is highly energy-demanding—particularly in osteoblasts, which require substantial ATP for collagen synthesis and matrix mineralization—GLP-1 acts as a critical metabolic regulator that enhances energy supply while counteracting metabolic stress. Central to this process is the induction of mitochondrial biogenesis: upon GLP-1 receptor activation, the cAMP/PKA pathway triggers AMPK phosphorylation, leading to upregulation of PGC-1α and TFAM.^163^ This signaling cascade promotes mitochondrial biogenesis and shifts osteoblasts from glycolysis toward oxidative phosphorylation, thereby meeting the high bioenergetic demands of mineralization. GLP-1 further supports osteoblast function by improving nutrient availability. Under insulin-resistant conditions, osteoblasts exhibit impaired glucose uptake; GLP-1 counteracts this by enhancing insulin sensitivity and promoting Glucose transporter 4 (GLUT4) translocation via the PI3K/Akt pathway, thus securing the carbon substrates necessary both for ATP production and collagen glycosylation.^164^^,^^165^ Additionally, GLP-1 mitigates metabolic insults in the bone marrow niche. It counteracts lipotoxicity and ferroptosis—an iron-dependent cell death process driven by lipid peroxidation—by activating the Nrf2/Glutathione peroxidase 4 (GPX4) antioxidant pathway, while also facilitating cholesterol efflux via ABCA1 upregulation.^111^^,^^166-168^ Together, these mechanisms illustrate how GLP-1 optimizes the intracellular metabolic milieu of bone cells, ensuring their functional integrity in the context of systemic metabolic challenges.

Although clinical data remain heterogeneous, emerging trends highlight GLP-1’s translational potential. Regarding fracture risk, several national and regional cohort studies (e.g., Danish National Registry, Xi’an Tangdu Hospital cohort) indicate that GLP-1RAs do not increase the risk of major osteoporotic fractures (MOF) when compared to other glucose-lowering agents such as dipeptidyl peptidase-4 (DPP-4) or sodium-glucose co-transporter-2 (SGLT2) inhibitors. In fact, they may reduce the risk of hip fractures. Only one Mendelian randomization study reported a modest positive association between GLP-1RAs use and osteoporotic fractures, potentially reflecting population or methodological differences.^148^^,^^149^^,^^169-171^ Concerning bone density and quality, treatment with dulaglutide or semaglutide for 12 months has been associated with mild reductions in femoral BMD (e.g., 4.1% decrease in total hip BMD), but trabecular bone score (TBS) remained stable or improved. Elevations in bone turnover markers (B-ALP, CTX) suggest increased remodeling activity without compromised bone quality. A small RCT in obese, non-diabetic patients taking antipsychotics further showed that short-term exenatide therapy could increase lumbar spine (L2–L4) BMD.^161^^,^^172^ At the genetic level, a case-control study in Chinese postmenopausal women identified associations between GLP-1R polymorphisms (e.g., rs1042044 A allele, rs3765468 AG/AA genotype) and susceptibility to postmenopausal OP. Certain SNP interactions (e.g., rs1042044 with rs2268641) and haplotypes (e.g., GACACA) were also correlated with increased risk.^150^^,^^151^ These findings, combined with GLP-1’s metabolic benefits and broad GLP-1R expression in bone tissue,^39^^,^^110^^,^^115^ support its clinical relevance in populations with OP complicated by diabetes or dyslipidemia.

The potent weight-loss effects of current GLP-1RAs have prompted legitimate concerns about skeletal integrity, particularly whether the associated mechanical unloading accelerates bone resorption and elevates fracture risk. Current evidence, however, reveals a distinction between physiological adaptations in bone density and long-term clinical outcomes. The reduction in BMD observed in trials such as STEP 1 likely reflects Wolff’s law—where bone adapts to reduced mechanical loading—rather than direct drug-related toxicity. In a STEP 1 sub-analysis, substantial weight loss (approximately 15%) was associated with declines in hip and spine BMD and elevated bone resorption markers such as CTX;^173^ yet, these changes appeared closely tied to weight loss itself. Importantly, during weight maintenance, GLP-1RAs demonstrate osteoprotective potential. For instance, Iepsen et al. reported that liraglutide maintained bone mineral content and increased the bone formation marker P1NP by 16%, countering the bone loss typically seen after dietary weight loss.^174^ Moreover, a 2024 randomized trial by Jensen et al. found that combining GLP-1RAs therapy with exercise fully preserved BMD at the hip and lumbar spine, despite greater weight loss than with pharmacotherapy alone—suggesting that mechanical loading can offset unloading-induced bone loss.^175^ Regarding fracture risk, long-term data indicate a neutral to protective skeletal safety profile. A 2025 retrospective cohort study of elderly patients with type 2 diabetes—a group at high risk for bone fragility—showed that long-term GLP-1RAs use was associated with a significantly lower incidence of OP (HR 0.69, 95% CI 0.45–0.84) compared with non-users.^170^ This is consistent with network meta-analyzes indicating that GLP-1RAs do not increase fracture rates, unlike some other glucose-lowering agents such as thiazolidinediones or canagliflozin.^176^ In OA, concerns about rapid joint space loss are not supported by longitudinal data. On the contrary, data from the Shanghai OA Cohort indicated that GLP-1RAs users experienced slower medial tibial cartilage volume loss and a significantly lower risk of progression to total knee arthroplasty.^144^^,^^177^ This structural preservation may result from both mechanical offloading and direct anti-inflammatory effects via GLP-1 receptor signaling, which help reduce catabolic breakdown of the extracellular matrix. In summary, although rapid weight loss warrants bone health monitoring in at-risk groups such as postmenopausal women with low baseline BMD,^174^ collective evidence supports the long-term skeletal safety of GLP-1RAs. Their pharmacologic profile appears intrinsically anabolic or neutral with respect to bone, accompanied by low fracture risk and potential structural benefits in OA.

Emerging observational data, particularly from real-world studies, suggest a threshold effect in the relationship between GLP-1RAs dosage and fracture risk. A Danish nationwide cohort study by Al-Mashhadi et al. found that while overall fracture risk reduction among GLP-1RAs users was not significant, higher cumulative daily doses were associated with a notably lower risk of hip fractures, whereas lower doses showed no such effect.^148^ This indicates that stronger activation of GLP-1 receptors may be needed to produce osteogenic activity capable of counteracting bone loss related to weight reduction. Liraglutide offers a relevant example: although standard glucose-lowering doses had neutral effects on BMD,^178^ the higher 3.0 mg dose used for obesity appeared to mitigate weight-loss-induced bone loss when combined with exercise.^174^^,^^175^ In contrast, semaglutide 2.4 mg was not linked to increased fracture risk but primarily induced bone resorption changes consistent with weight loss, with less clear evidence of direct bone-forming effects.^173^^,^^179^ Thus, higher GLP-1RAs doses, particularly of liraglutide, may offer greater skeletal benefit by promoting anabolic bone activity that helps offset resorptive effects of weight loss. At the same time, these skeletal benefits appear time-dependent, becoming evident only after sustained treatment. A meta-analysis by Cheng et al. indicated that studies shorter than about 52 weeks showed no fracture risk reduction, while longer studies did,^179^ which aligns with the slow timeline of bone remodeling and microarchitectural improvement. The long-term skeletal safety of GLP-1RAs is supported by major cardiovascular outcome trials reporting neutral fracture risk over several years of follow-up.^38^^,^^180^ However, discontinuation of therapy may compromise skeletal gains due to weight rebound and potential shifts toward sarcopenic obesity, highlighting the importance of continued treatment.^181^ It should also be noted that rapid weight loss from GLP-1RAs may reduce BMD. Therefore, clinical guidance should emphasize long-term management: patients, especially those at high fracture risk, should be informed that fracture protection benefits may require at least one year of therapy and are best sustained through long-term treatment combined with resistance exercise to support metabolic and musculoskeletal health.

Collective evidence from cell, animal, and clinical studies underscores the therapeutic potential of GLP-1 and GLP-1RAs in OP. By promoting osteoblastogenesis and suppressing osteoclast-mediated resorption, GLP-1 addresses the core imbalance in bone metabolism. Nevertheless, clinical application faces challenges such as undefined indications, limited mechanistic validation, and inconsistent endpoints. Future research should prioritize large-scale, multicenter, mechanism-driven clinical trials in primary OP populations to establish cross-level translational evidence and facilitate the integration of GLP-1 into routine OP management.

#### 3.2.3 Specific role of GLP-1 in SP

Unlike other degenerative musculoskeletal disorders, the pathological hallmark of SP lies in a dual imbalance: impaired myogenesis and exacerbated muscle atrophy. The therapeutic potential of GLP-1 in SP stems from its precise regulatory effects on both muscle regeneration and atrophy-related signaling pathways. On the one hand, GLP-1 enhances muscle regenerative capacity by upregulating key myogenic transcription factors such as MyoD and MyoG, thereby promoting myotube formation and differentiation. On the other hand, it suppresses the overexpression of atrophy-inducing genes—such as Atrogin-1, MuRF-1, and myostatin (MSTN)—inhibiting protein degradation mediated by the ubiquitin–proteasome system (UPS) and preserving muscle fiber size and functional stability. Additionally, GLP-1 contributes to mitochondrial biogenesis and improves energy metabolism, including glucose and lipid utilization, thus providing both structural and metabolic support for muscle function. This integrated mechanism of action—comprising “pro-myogenesis, anti-atrophy, and energy stabilization”—directly corresponds to the central pathological triad of SP, and distinguishes GLP-1 from therapeutic strategies targeting bone or cartilage degeneration in other musculoskeletal diseases.

Cell-based studies, primarily using the C2C12 murine myoblast model, have revealed multiple regulatory roles of GLP-1 in myogenesis and muscle atrophy. GLP-1RAs (e.g., semaglutide, liraglutide) have been shown to reverse palmitic acid- or high glucose-induced inhibition of myogenesis, promote myotube formation and elongation, and upregulate myogenic markers such as MyoD, MyoG, and MyHC isoforms. Concurrently, they inhibit the expression of atrophy-related markers (Atrogin-1, MuRF-1, MSTN) and reduce the proportion of senescence-associated *β*-galactosidase (SA-*β*-gal)-positive senescent cells. Mechanistically, these effects are mediated via the cAMP/PKA-HSF1 signaling axis.^119^ Other studies suggest involvement of the YAP–TAZ pathway in restoring Cyclin D1 expression,^120^ and the SIRT1 signaling axis in enhancing mitochondrial biogenesis.^118^ Additional GLP-1RAs, including Exendin-4, PF1801, and Dulaglutide, also activate GLP-1R signaling, facilitate myogenic differentiation, and upregulate myogenic gene expression (e.g., MyoD, MyoG, MyHC4/7) and myokines (e.g., FNDC5). Exendin-4 specifically enhances glucose uptake by promoting GLUT4 translocation, upregulates glycogen synthesis genes (Gys1, Gys2), and increases mitochondrial DNA content and respiratory capacity.^40^ Dulaglutide activates AMPK to induce heat shock protein 72 (Hsp72) and upregulate mitochondrial PGC-1α.^116^^,^^182^ PF1801, via the AMPK–PGAM5 pathway, protects against FASLG-induced necroptosis in myotubes.^122^ Overall, these agents enhance mitochondrial function and energy availability, supporting muscle regeneration and functional maintenance.

GLP-1RAs have shown robust muscle-protective effects across various animal models of SP and muscle dysfunction. In diabetes-related models (e.g., KK-Ay mice, STZ-induced rats, db/db mice), GLP-1RAs improve muscle mass, increase gastrocnemius and psoas major muscle weight, and enlarge muscle fiber cross-sectional area (CSA). Improvements in functional parameters, such as grip strength (forelimb grip with Exendin-4, overall grip with Dulaglutide), and fast-twitch fiber preservation have been observed.^116^^,^^183^^,^^184^ These effects are associated with activation of the Silent Information Regulator 2 homolog 1 (SIRT1) and YAP–TAZ pathways, suppression of UPS-mediated degradation, and inhibition of atrophy-related gene expression. GLP-1RAs also improve metabolic comorbidities, such as liver and kidney function (e.g., lowering BUN) and insulin resistance, indirectly supporting muscle anabolism. Similar muscle-protective effects have been reported in obesity models (e.g., high-fat diet-induced SP),^117^^,^^183^ chronic liver disease models (e.g., DDC-fed mice),^118–120^ and immune myositis models (e.g., C-protein-induced myositis), where PF1801 alone or with prednisone reduces muscle necrosis and restores CSA.^122^ In disuse atrophy models, Dulaglutide activates the AMPK–Hsp72 pathway to suppress apoptosis-related proteins (e.g., caspase-3, cleaved PARP), thereby preserving muscle mass.^182^ In Duchenne muscular dystrophy models, Exendin-4 and Dulaglutide improve CSA, prolong hanging time, and upregulate myogenic markers.^116^ Notably, GLP-1 overexpression can shift muscle fiber composition by increasing type I (slow-twitch) fibers and decreasing type IIb/x (fast-twitch) fibers in the gastrocnemius, structurally enhancing endurance capacity.^40^ A key challenge in translating preclinical findings to humans is the species-specific distribution of the GLP-1R. While rodent studies consistently demonstrate that GLP-1RAs directly promote a shift from fast-twitch (Type IIb/x) to slow-twitch (Type I) muscle fibers via AMPK–SIRT1–PGC-1α signaling within myocytes,^118^^,^^135^^,^^185^ the direct expression of GLP-1R in human skeletal muscle cells remains incompletely established.^186^ Transcriptomic and histological evidence indicates that functional GLP-1R is predominantly localized to vascular endothelial and smooth muscle cells.^187-189^ Therefore, rather than direct genomic reprogramming of myosin heavy chain (MHC) isoforms as observed in animals,^190^ the human “fiber-type transition” likely represents a functional oxidative remodeling mediated through the vascular system.^187^ This indirect mechanism operates primarily via GLP-1-induced microvascular recruitment, which enhances capillary perfusion and oxygen delivery to active muscle tissue.^188^^,^^191^ By alleviating hypoxia—a common feature in obesity and insulin resistance—GLP-1RAs create a physiological environment favorable for oxidative metabolism, thereby simulating the functional characteristics of Type I fibers. Additionally, reduced myosteatosis (ectopic lipid accumulation) improves mitochondrial coupling efficiency, further reinforcing an endurance-adapted phenotype.^191^ This mechanistic distinction carries important clinical implications: although patients exhibit improved endurance—as reflected by increased 6-minute walk distance in trials such as STEP-HFpEF—absolute muscle strength remains susceptible to decline.^192^ Since GLP-1 signaling does not directly activate anabolic pathways such as mTOR in human muscle, the catabolic state induced by caloric restriction may promote atrophy of glycolytic Type II fibers.^142^ Thus, although GLP-1RAs enhance metabolic fitness and fatigue resistance, preserving muscle strength and power likely requires concurrent resistance training to offset the lack of direct pharmacological anabolism.

Observational studies reveal that circulating GLP-1 levels are elevated in SP populations—particularly among older adults and individuals with type 2 diabetes—and are associated with declines in fat-free mass (FFM) and appendicular skeletal muscle mass (ASM).^17^^,^^127^^,^^193^ Interventional trials demonstrate that GLP-1RAs, alone or in combination with basal insulin, can maintain or modestly increase ASM and FFM in elderly patients with type 2 diabetes, while reducing fat mass and body fat percentage. No significant association with SP incidence has been reported. Some studies suggest that higher doses of GLP-1 may have additional metabolic effects on muscle tissue.^141^^,^^194^^,^^195^ For example, in overweight or obese elderly patients with type 2 diabetes, a 24-week liraglutide treatment maintained skeletal muscle index (SMI), reduced fat mass, and prevented SP onset.^196^ Muscle biopsy samples from patients with polymyositis or dermatomyositis revealed elevated GLP-1R expression localized to inflamed fibers and sarcolemma, implying a role for GLP-1 signaling in muscle repair and regeneration in inflammatory myopathies.^122^

GLP-1, through its multifaceted mechanism of “promoting myogenesis, resisting atrophy, and stabilizing energy metabolism,” aligns precisely with the pathological features of SP, as validated by evidence from cellular studies, animal models, and clinical research. This unique mode of intervention not only emphasizes the direct regulation of muscle fiber regeneration and metabolic function but also enhances systemic energy homeostasis via the muscle–liver–pancreas axis. The therapeutic advantage of GLP-1 in SP, distinct from its matrix-targeting mechanisms in OP and OA, highlights its unique clinical value in the context of SP.

#### 3.2.4 Specific role of GLP-1 in IVDD

Unlike other degenerative musculoskeletal disorders, the pathological core of IVDD lies in the vicious cycle of NPCs injury and disruption of ECM homeostasis. The therapeutic efficacy of GLP-1 in this context relies primarily on its direct regulatory effects on NPCs and its precise modulation of ECM synthesis and degradation, constituting a unique advantage in IVDD intervention. On one hand, GLP-1 enhances NPCs viability by modulating cell fate decisions; on the other, it preserves the structural integrity of the nucleus pulposus by promoting ECM production and suppressing degradative enzyme activity. This dual mechanism directly targets the central pathological axis of IVDD. The integrated intervention strategy of “anti-injury and matrix preservation” aligns closely with the key pathological hallmark of IVDD—NPCs injury and ECM degradation—and distinguishes GLP-1 from therapeutic approaches used in other degenerative musculoskeletal conditions.

At the cellular level, the protective effects of GLP-1 on NPCs have been consistently attributed to GLP-1R-dependent mechanisms. Inhibition of GLP-1R via the antagonist Exendin (9-39)^123^ or gene silencing using siRNA^128^ significantly attenuates these effects, confirming the necessity of GLP-1R signaling. Furthermore, human NPCs have been shown to express high levels of GLP-1R, providing a molecular basis for therapeutic targeting in human tissue. GLP-1RAs have demonstrated the ability to attenuate NPCs damage induced by various pathological stimuli. For example, exenatide dose-dependently reduces ROS accumulation in both rat and human NPCs exposed to tert-butyl hydroperoxide (TBHP), a model of oxidative stress. Liraglutide significantly decreases apoptosis rates in human NPCs under high-glucose conditions.^123^^,^^128^ Beyond their established metabolic effects, GLP-1 receptor agonizts exert therapeutic benefits in IVDD by fundamentally reprogramming the transcriptional activity of degenerating NPCs. A central mechanism underlying this protection involves the selective modulation of Activator Protein-1 (AP-1). Under hypoxic and inflammatory conditions characteristic of IVDD, upstream MAPK signaling—particularly via JNK and p38—becomes hyperactive, driving the assembly of AP-1 dimers that bind to TPA-response elements (TREs) in catabolic gene promoters.^197–201^ Rather than broadly suppressing AP-1, GLP-1 specifically impedes the formation of basic leucine zipper ATF-like transcription factor (BATF)/Jun proto-oncogene (JUN) heterodimers, which—unlike AP-1 homodimers—serve as principal drivers of MMP13 and ADAMTS5 transcription.^197^^,^^199^ Through this targeted disruption, GLP-1 mitigates the enzymatic degradation of collagen II and aggrecan, thereby preserving extracellular matrix integrity.^123^^,^^202^ In addition to AP-1 regulation, GLP-1 orchestrates a multi-faceted defense that encompasses NF-κB and signal transducer and activator of transcription 3 (STAT3) signaling pathways. Concurrently with AP-1 inhibition, GLP-1 curtails NF-κB activation by enhancing SIRT1, which deacetylates the RelA/p65 subunit and limits its nuclear translocation, leading to suppression of IL-1β and TNF-*α* expression.^202-204^ Furthermore, GLP-1 differentially modulates STAT3 in a cell type–specific manner: it attenuates pathological STAT3 phosphorylation in NPCs to reduce cellular senescence, while potentiating STAT3 signaling in infiltrating macrophages to promote a pro-regenerative M2 polarization, thereby fostering an immune microenvironment conducive to tissue repair.^200^^,^^202^^,^^205^ Collectively, these findings establish GLP-1 as a key transcriptional regulator that simultaneously targets metabolic, inflammatory, and catabolic processes to decelerate disc degeneration.

In vivo validation using a rat caudal intervertebral disc puncture model of IVDD has corroborated the findings from *in vitro* experiments. Intradiscal injection of exenatide significantly improved degenerative pathology. Histological analysis via hematoxylin–eosin (H&E) and Safranin-O staining revealed increased cell viability, enhanced proteoglycan content, and more uniform ECM distribution. MRI T2-weighted imaging further demonstrated increased nucleus pulposus hydration and signal intensity—hallmarks of preserved disc structure. These improvements were accompanied by reduced ROS deposition and suppressed expression of BATF, JUNB, and JUNC, indicating lowered AP-1 activity. Importantly, BATF overexpression reversed these protective effects, providing strong mechanistic validation consistent with cellular-level findings.^123^

The compelling rationale for GLP-1RAs in degenerative disc disease is deeply rooted in their capacity to counteract the core pathological triad of IVDD—nutrient deprivation, oxidative stress, and meta-inflammation—through mechanisms that both converge with and diverge from their actions in OA and OP. In IVDD, the avascular nucleus pulposus presents a unique metabolic challenge where GLP-1 signaling provides crucial cytoprotection via PI3K/Akt/mTOR pathway activation, directly combating the apoptosis that drives disc collapse, while simultaneously modulating autophagic flux to maintain cellular homeostasis in this harsh microenvironment.^135^ This aligns conceptually with its chondroprotective role in OA, where it similarly suppresses NF-κB-driven catabolism and matrix degradation,^133^ yet the IVDD rationale is further distinguished by the emerging understanding of its potential to regulate mitochondrial quality control and mitigate ER stress.^206-208^ Conversely, the skeletal rationale in OP operates on a fundamentally different axis of tissue remodeling, pivoting on the anabolic Wnt/β-catenin activation in osteoblasts and the critical rebalancing of the OPG/RANKL ratio,^137^^,^^209^^,^^210^ mechanisms largely irrelevant to the post-mitotic, matrix-rich tissues of the disc and joint. Thus, while the gut-joint/disc/bone axis represents a unified paradigm of systemic meta-inflammatory signaling, the tissue-specific manifestation of GLP-1 efficacy reveals a sophisticated biological narrative: it functions as a direct cellular guardian in the mechanically stressed, aneural environments of the disc and cartilage, but as a master regulator of coupled cellular turnover in the dynamic organ that is bone.

Beyond functioning in isolation through the GLP-1 receptor, GLP-1 can act synergistically with other metabolic regulators such as fibroblast growth factor 21 (FGF21) and adiponectin. A growing body of preclinical and emerging clinical evidence supports the presence of such synergy. Physiologically, activation of the GLP-1 receptor upregulates hepatic sensitivity to FGF21 and stimulates adiponectin secretion from adipose tissue,^211-213^ establishing a positive feedback loop that enhances systemic insulin sensitivity. Pharmacologically, novel dual agonizts—for instance, GLP-1/FGF21 chimeric peptides—exhibit superior efficacy over monotherapies in modulating lipid metabolism and energy expenditure,^214^ largely through integrated downstream signaling via the AMPK pathway.^215^^,^^216^ In the context of SP, this combination counteracts the loss of lean mass frequently observed during rapid weight loss: GLP-1 promotes insulin-mediated protein synthesis,^191^ while FGF21 enhances mitochondrial quality control and fatty acid oxidation.^217^ Together, they alleviate intramuscular lipotoxicity and anabolic resistance, thereby preserving muscle integrity during metabolic adaptation. With regard to IVDD, the synergistic mechanism may address the unique avascular nature of the disc. While GLP-1 exerts systemic glycemic control and attenuates high-glucose-induced apoptosis,^218^ FGF21 and adiponectin act locally to restore autophagic flux and suppress NF-κB-driven inflammation.^219^^,^^220^ This dual “systemic-plus-local” targeting offers a more comprehensive strategy to impede the progression of diabetic disc degeneration compared to single-agent approaches.

Current evidence indicates that long-term exposure to GLP-1 receptor agonizts does not lead to maladaptive receptor downregulation or the development of pharmacological resistance in skeletal muscle or intervertebral disc tissues. Instead, tissue-specific mechanisms appear to sustain their efficacy. In skeletal muscle, where GLP-1 receptor expression in human myocytes remains a subject of debate,^142^^,^^221^ the reduction in lean mass observed clinically is better explained as a physiological adaptation to negative energy balance—reflecting thermodynamic repartitioning—rather than as a form of drug tolerance.^142^^,^^222^^,^^223^ Importantly, the primary beneficial mechanism in this tissue, namely the enhancement of microvascular perfusion mediated by endothelial GLP-1 receptors, remains effective during chronic therapy without signs of tachyphylaxis.^185^^,^^224-226^ In intervertebral disks, GLP-1RAs exhibit what may be described as a “receptor rescue” effect. Although degenerative and hyperglycemic conditions pathologically suppress GLP-1 receptor expression in NPCs,^227^ GLP-1RAs treatment counteracts this by inhibiting NF-κB-mediated transcriptional repression, thereby restoring and maintaining receptor levels.^42^^,^^128^ This maintained signaling capacity is consistent with long-term clinical data showing a progressive reduction in the need for lumbar spine surgery over a 5-year period, supporting the view that therapeutic efficacy is preserved—and not diminished—over time.^228^

Current translational and clinical evidence supports the combined use of GLP-1RAs with established standard-of-care interventions for both SP and IVDD, pointing to synergistic mechanisms that go beyond simple additive effects. In SP, contrary to concerns about catabolic risk, GLP-1RAs appear to enhance the benefits of physical therapy and nutritional support through hemodynamic modulation. Age-related “anabolic resistance”—a blunted muscle protein synthesis response to nutrient intake—is partly attributable to impaired capillary perfusion. GLP-1 infusion has been shown to increase skeletal muscle microvascular blood volume by approximately 30–40% via a nitric oxide-dependent pathway. This vasodilation acts as a physiological gatekeeper, improving delivery of amino acids and insulin to myocytes.^185^^,^^229^^,^^230^ When combined with resistance training and high-protein intake (>1.2 gram [g]/kg/day), GLP-1RAs may thus help preserve muscle mass despite weight loss.^231^ In the context of IVDD, glucocorticoids—though effective for acute radiculopathy—promote osteoblast apoptosis and suppress bone formation, increasing risks of OP and osteonecrosis. Preclinical models indicate that GLP-1RAs such as liraglutide and exenatide preserve mitochondrial integrity in bone cells exposed to dexamethasone.^128^ This supports the potential use of GLP-1RAs as bone-protective adjuvants during chronic steroid therapy. Concurrent use of GLP-1RAs and NSAIDs offers theoretical synergy through dual inhibition of inflammatory pathways—targeting NF-κB signaling and COX activity,^232^ respectively—but requires careful renal monitoring due to increased risk of acute kidney injury. GLP-1RAs–induced volume loss, combined with NSAIDs-mediated afferent arteriolar vasoconstriction and concurrent renin–angiotensin system inhibition, creates a “triple whammy” effect that compromises renal perfusion.^233^^,^^234^ Finally, retrospective cohort studies indicate that perioperative GLP-1RAs administration is associated with a lower incidence of pseudarthrosis after lumbar fusion, likely due to the mitigation of hyperglycemia—which inhibits osteogenesis—and direct stimulation of osteoblast differentiation, highlighting a potential role for GLP-1RAs in supporting surgical repair.^235^^,^^236^

GLP-1, through its dual regulatory roles in “injury attenuation” and “ECM preservation,” offers a novel and targeted intervention strategy that aligns precisely with the pathophysiological features of IVDD. Although clinical validation is currently lacking, existing preclinical evidence has demonstrated its unique therapeutic potential—distinct from interventions targeting bone or cartilage matrix in OP and OA. These findings lay a solid theoretical and experimental foundation for future clinical translation and highlight GLP-1 as a promising candidate in the treatment of IVDD.

## 4 Intervention strategies targeting the “GM–GLP-1” axis to treat degenerative musculoskeletal diseases

### 4.1 Clinical and mechanistic rationale for GLP-1–based therapy in degenerative musculoskeletal diseases

The regulatory landscape for GLP-1RAs has undergone a fundamental shift from a predominantly glucocentric approach to one centered on comprehensive organ protection. By early 2025, the U.S. Food and Drug Administration (FDA) and European Medicines Agency (EMA) had expanded the approved uses of these agents to reflect their pleiotropic effects on metabolic, cardiovascular, and renal systems.^237^ Current indications cover four key areas: type 2 diabetes, obesity, cardiovascular risk reduction, and chronic kidney disease. In clinical practice, GLP-1 receptor agonizts and dual glucose-dependent insulinotropic polypeptide (GIP)/GLP-1 agonizts are now used not only for glucose control but also for weight loss, cardiovascular protection, and renal outcomes.^237^^,^^238^ For example, the Semaglutide Effects on Cardiovascular Outcomes in People with Overweight or Obesity (SELECT) trial demonstrated that semaglutide significantly reduces major adverse cardiovascular events in patients with established cardiovascular disease (CVD), regardless of diabetes status.^239^^,^^240^ Similarly, high-dose semaglutide and tirzepatide have shown weight loss efficacy close to that of bariatric surgery, supporting their approval in obesity;^241^^,^^242^ and chronic kidney disease, where results from the Semaglutide and Renal Outcomes in Patients with Type 2 Diabetes and Chronic Kidney Disease (FLOW) trial led to the approval of semaglutide in early 2025 for slowing renal progression in patients with type 2 diabetes and chronic kidney disease (CKD). Underpinning these indications are large-scale outcome trials that elucidate mechanisms beyond glucose-lowering.^243^ In cardiovascular outcomes, the SELECT trial revealed a 20% reduction in MACE with semaglutide in non-diabetic obese patients with CVD, an effect largely attributed to direct vascular and anti-inflammatory actions rather than weight loss alone.^239^^,^^244^ Similarly, in renal protection, the FLOW trial demonstrated a 24% risk reduction in a composite renal endpoint with semaglutide, positioning GLP-1RAs alongside SGLT2 inhibitors as standard care in diabetic kidney disease.^245^^,^^246^ Beyond metabolic and cardio-renal domains, emerging evidence points to potential disease-modifying effects of GLP-1RAs in degenerative musculoskeletal conditions, likely mediated through attenuation of chronic meta-inflammation. In knee OA, the STEP 9 trial reported superior pain reduction with semaglutide compared to placebo, exceeding improvements attributable to weight loss alone and suggesting direct anti-nociceptive and synovial anti-inflammatory activity.^145^ Concerns over sarcopenic obesity with GLP-1RAs use have prompted investigation into combination therapies;^247^^,^^248^ the phase 2 Bimagrumab and Semaglutide for Body Composition in Obesity (BELIEVE) trial showed that adding bimagrumab to semaglutide enhanced fat loss while increasing lean mass, offering a “quality weight loss” strategy particularly relevant in aging populations.^248^^,^^249^ Furthermore, although weight loss typically elevates bone turnover, meta-analyzes indicate no increased fracture risk with GLP-1RAs, and some data suggest protective effects on lumbar BMD.^173^^,^^174^ Preclinical and retrospective clinical studies also imply that GLP-1RAs may slow IVDD, potentially through activation of PI3K/Akt/mTOR signaling and suppression of oxidative stress-induced apoptosis in NPCs, pointing to novel therapeutic avenues in chronic low back pain.^128^^,^^250^^,^^251^

### 4.2 Targeting the GM–GLP-1 axis: from optimizing pharmacotherapy to preventing degenerative musculoskeletal diseases

A compelling avenue for optimizing GLP-1RAs therapy lies in its combination with GM-directed interventions. This strategy is supported by a growing body of evidence demonstrating both the mitigation of treatment-limiting side effects and the enhancement of therapeutic efficacy. The high prevalence of gastrointestinal adverse events, such as nausea, constipation, and diarrhea, frequently impedes dose escalation and long-term adherence.^252^^,^^253^ Clinical studies, including the ongoing trial NCT07213323, are explicitly investigating the hypothesis that specific probiotic formulations can improve GI tolerability during the GLP-1RAs dose-titration phase. This is mechanistically supported by the known actions of probiotic strains; for instance, *Bifidobacterium lactis BB-12* can ameliorate constipation by improving intestinal transit, while *Lactobacillus acidophilus NCFM* can modulate visceral hypersensitivity, potentially reducing nausea.^254-256^ Beyond managing side effects, this combination offers a synergistic boost to metabolic outcomes. The gut microbiome serves as a potent endogenous modulator of GLP-1 secretion. Microbial fermentation of dietary fibers produces SCFAs, which activate specific receptors (FFAR2/3) on L cells to stimulate the release of endogenous GLP-1 and Peptide YY (PYY).^31^ Furthermore, GLP-1RAs have been shown to increase the abundance of beneficial species like *Akkermansia muciniphila*, which in turn secretes bioactive proteins (e.g., P9) that further stimulate GLP-1 secretion.^257^^,^^258^ This creates a positive feedback loop, where pharmacological intervention and microbial ecology work in concert to amplify the GLP-1 signal, potentially leading to superior glycemic control and weight management. Importantly, this synergistic approach addresses an emerging concern regarding GLP-1RAs-induced rapid weight loss: the risk of BMD loss and SP. Through the 'gut-bone axis,' prebiotics like inulin enhance mineral absorption and SCFAs suppress bone resorption by modulating immune cells.^259^^,^^260^ Concurrently, via the 'gut-muscle axis,' certain probiotics (e.g., *Lactobacillus plantarum*) have been shown to activate anabolic signaling pathways (e.g., mTOR) and improve mitochondrial function in skeletal muscle, thereby counteracting muscle wasting.^261^^,^^262^ Therefore, the combination therapy not only aims to improve the primary metabolic endpoints but also introduces a novel dimension of protecting musculoskeletal health during significant weight loss.

The established role of the GM–GLP-1 axis in regulating skeletal and muscle homeostasis leads to a key hypothesis: its targeted modulation could represent not only an adjunct to GLP-1RAs therapy but a foundational strategy for a new, integrative intervention against degenerative musculoskeletal diseases.

**Figure 4.**
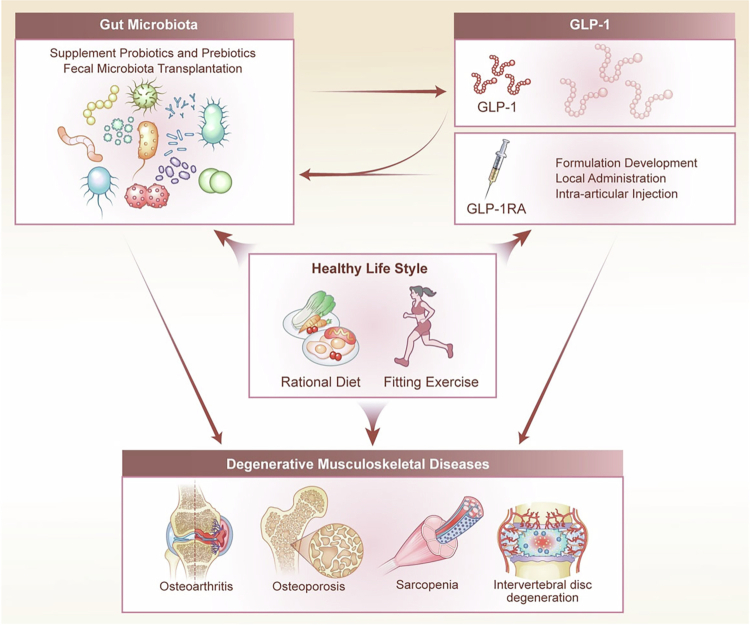
Intervention strategies targeting the “GM–GLP-1” axis to treat various degenerative musculoskeletal diseases can be implemented through multiple approaches. First, healthy lifestyle factors—such as a rational diet and fitting exercise—help modulate the GM, enhance GLP-1 levels, and slow the progression of degenerative musculoskeletal diseases, including osteoarthritis, osteoporosis, sarcopenia, and intervertebral disc degeneration. These three effects (microbiota modulation, GLP-1 elevation, and slowing disease progression) are closely linked: the modulation of the GM acts as a potential upstream driver for increased GLP-1 levels, and together they contribute to alleviating the pathological processes of degenerative musculoskeletal diseases. Second, direct modulation of the GM is another key strategy. This can be achieved through supplements such as probiotics and prebiotics, or therapeutic approaches like FMT, all of which can influence GLP-1 secretion and impact the progression of these diseases. Notably, GLP-1 and GLP-1RAs serve as central mediators in this axis, playing critical dual roles: they not only modulate GM homeostasis but also regulate the pathological processes underlying degenerative musculoskeletal conditions. Furthermore, targeted GLP-1 signaling interventions have been developed to optimize therapeutic efficacy, including novel GLP-1 formulations, localized administration, and intra-articular injections. These approaches directly target GLP-1 signaling pathways to enhance musculoskeletal health, while also reciprocally modulating the GM—forming a synergistic regulatory loop that amplifies the intervention effect on degenerative musculoskeletal diseases. GM: gut microbiota; GLP-1: glucagon-like peptide-1; GLP-1RAs: GLP-1 receptor agonizts.

The onset and progression of degenerative musculoskeletal diseases are closely associated with systemic chronic low-grade inflammation and metabolic dysregulation. The intricate crosstalk between the GM and GLP-1 offers a novel perspective for the prevention and treatment of these conditions (Figure 4). Growing evidence indicates that regular physical activity^263^^,^^264^ and a well-balanced diet^265^^,^^266^ can directly modulate the composition of the GM and the production of its metabolites. These lifestyle factors concurrently regulate systemic energy balance and inflammation, thereby influencing GLP-1 secretion. A healthy lifestyle thus represents a common foundation that simultaneously improves GM composition, enhances GLP-1 release, and attenuates the pathological progression of degenerative musculoskeletal diseases.^267^^,^^268^ Based on this rationale, we propose an integrated intervention strategy targeting the “GM–GLP-1” axis to treat various degenerative musculoskeletal diseases. The initial stage of intervention focuses on the direct modulation of the gut microecological environment. Among various approaches, the supplementation of probiotics and prebiotics remains the most accessible and widely applied approaches.^269–271^ Administration of specific probiotic strains (e.g., *Lactobacillus*, *Bifidobacterium*) increases the abundance of beneficial intestinal flora, while prebiotics such as inulin and fructooligosaccharides serve as substrates for their growth and proliferation.^272^ Improved GM composition enhances intestinal barrier integrity and reduces the translocation of pro-inflammatory mediators into the circulation, thereby mitigating systemic inflammatory responses implicated in musculoskeletal degeneration. Moreover, the fermentation of dietary fibers by commensal bacteria leads to the production of SCFAs and other metabolites, which in turn stimulate intestinal L cells to secrete GLP-1. This cascade not only reinforces metabolic homeostasis but also initiates GLP-1-mediated anti-inflammatory and tissue-protective pathways. In cases of severe dysbiosis, FMT may offer a more robust means to re-establish a healthy gut ecosystem and restore functional microbial balance.^273^ GLP-1RAs occupy a central position in this therapeutic paradigm by serving as both effectors and modulators of the GM–GLP-1 axis. Pharmacologically, GLP-1RAs mimic the endogenous functions of GLP-1, exerting systemic anti-inflammatory effects, promoting bone health, and preventing muscle atrophy—thereby offering therapeutic benefits across various degenerative musculoskeletal diseases. Simultaneously, recent studies suggest that GLP-1RAs themselves can influence gut microbial composition.^16^ By altering bile acid metabolism and delaying gastrointestinal transit, they facilitate the proliferation of beneficial bacterial populations and enhance overall microbial diversity. This bidirectional interaction creates a positive feedback loop in which GLP-1RAs not only exert therapeutic effects but also improve the underlying GM environment, thereby potentiating their own efficacy. To further optimize the therapeutic efficacy and specificity of GLP-1RAs in the treatment of degenerative musculoskeletal conditions, advanced drug delivery systems are critical. Given the need for long-term treatment, the development of long-acting or sustained-release oral formulations can improve patient adherence and maintain consistent therapeutic plasma concentrations. However, for localized conditions such as OA, systemic administration may be suboptimal. In such cases, targeted intra-articular delivery of GLP-1RAs directly into the affected joint may achieve higher local drug concentrations, enabling effective suppression of inflammation and preservation of cartilage integrity while minimizing systemic exposure and associated side effects. This localized strategy holds promise for achieving precision therapy. From foundational interventions such as dietary modulation and exercise to gut microecological optimization, GLP-1 pathway activation, and formulation-targeted drug delivery, a systematic, multi-level intervention framework is emerging for degenerative musculoskeletal diseases. This GM–GLP-1–targeted therapy axis offers considerable translational potential. Future research should focus on exploring this axis in greater depth, particularly in areas such as personalized medicine, combination therapy regimens, and mechanistic elucidation. Such efforts are essential to fully realize the clinical potential of this integrative therapeutic strategy.

## 5 Conclusion and perspective

In summary, GLP-1 exhibits multifaceted therapeutic potential across a spectrum of degenerative musculoskeletal diseases, including OA, OP, SP, and IVDD. By precisely targeting key pathological processes—such as cartilage degradation, bone metabolic imbalance, muscle atrophy, and NPCs injury—GLP-1 exerts a range of protective effects through mechanisms involving anti-inflammatory activity, matrix preservation, energy homeostasis, and regulation of cell fate. Notably, the emerging interplay between GM and GLP-1 adds a new dimension to this therapeutic paradigm. Lifestyle interventions, microbiota-targeted strategies (e.g., probiotics, prebiotics, FMT), and GLP-1RAs collectively form a regulatory network capable of restoring systemic and local musculoskeletal homeostasis.

Notwithstanding these promising findings, translating them into clinical practice requires overcoming several key challenges. A central issue is the heterogeneity of disease subtypes and patient populations; future studies must therefore examine differential responses to GLP-1 pathway modulation across distinct endotypes of OA, OP, SP, and IVDD—a necessary step toward personalized therapeutic approaches. Moreover, the precise mechanisms by which the complex, dynamic alterations in GM influence GLP-1 secretion and receptor expression remain unclear. Equally critical is the current lack of clinically applicable biomarkers, highlighting the urgency to develop and validate non-invasive tools capable of reporting GLP-1 pathway activity along the gut–musculoskeletal axis to support patient stratification and treatment monitoring. To address these gaps, we propose several research priorities: first, the design of multicenter, interdisciplinary clinical studies focusing on the GM–GLP-1–musculoskeletal axis in well-phenotyped cohorts, incorporating deep microbial sequencing, metabolomics, and advanced imaging; second, mechanistic studies using human tissue biorepositories and sophisticated animal models to clarify the molecular basis of GM–GLP-1 crosstalk; and finally, innovations in drug delivery—such as sustained-release formulations or localized administration of GLP-1RAs—to improve efficacy and reduce systemic effects. Ultimately, GLP-1 acts not only as a therapeutic agent but also as a central node linking GM, metabolic homeostasis, tissue repair, and immune regulation. Addressing these priorities will help catalyze a shift toward mechanism-based, microbiota-informed precision medicine for degenerative musculoskeletal diseases.

## Data Availability

Data sharing is not applicable to this article as no new data were created or analyzed in this study.
